# Quantitative Host Cell Protein Analysis of Antibody-Based Protein Therapeutics Using the Orbitrap Astral Mass Spectrometer

**DOI:** 10.1021/jasms.5c00272

**Published:** 2026-04-17

**Authors:** Josh Smith, Aaron Richardson, Corentin Beaumal, Marina Ainciburu, Sara Carillo, Anna Pashkova, Tabiwang N. Arrey, Nicolaie E. Damoc, Colin Clarke, Jonathan Bones

**Affiliations:** † 162828 National Institute for Bioprocessing Research and Training, Foster Avenue, Mount Merrion, Blackrock, Co., Dublin A94 X099, Ireland; ‡ Thermo Fisher Scientific GmbH, Hannah-Kunath Strasse 11, 28199 Bremen, Germany; § School of Chemical and Bioprocess Engineering, University College Dublin, Belfield, Dublin 4 D04 V1W8, Ireland

**Keywords:** host cell proteins, mAb based therapeutics, Orbitrap Astral MS, high risk HCPs, Biophorum, LC-MS/MS

## Abstract

Host cell proteins (HCPs) are endogenous proteins generated in cellular production systems alongside the biotherapeutic of interest. Removal of HCPs is crucial as they can be detrimental to product efficacy and patient safety. Due to its ability to determine individual HCP concentrations, liquid chromatography tandem mass spectrometry is increasingly utilized as an orthogonal method to ELISA for HCP monitoring. For protein biotherapeutics like monoclonal antibodies, their dynamic range makes detection of low-level HCPs difficult. The Orbitrap Astral MS has the potential to overcome such challenges, offering improvements in protein identifications in complex sample matrices while simultaneously reducing analysis times. Here, we utilize the Orbitrap Astral MS to perform HCP analysis on 36 protein biotherapeutics. Our workflow used a short 60 samples-per-day separation method and was initially benchmarked against four previously published studies, demonstrating comparable levels of HCP identifications. 236 HCPs were detected across the cohort and 55% of those found to be quantifiable in at least one product using label free quantitation. Functional analysis revealed that most detected HCPs had functions related to catalysis or binding, predominately catalytic activity (46%, 97 gene IDs) or protein binding (44%, 91 gene IDs). Nearly 80% of quantifiable HCPs were detected at concentrations below 10 ng/mg, with 8% detected below concentrations of 1 ng/mg. These included HCPs considered as “high-risk” by the Biophorum Development Group. This study shows how new generation mass spectrometry instruments can enable detection of low-level HCPs while allowing for a rapid and more informed understanding of a product’s HCP content.

## Graphical abstract

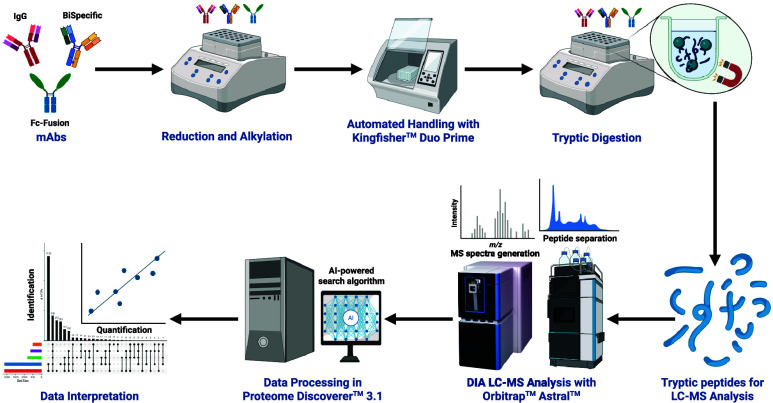

## Introduction

Recombinant protein biotherapeutics, particularly monoclonal antibodies (mAbs), constitute the significant part of biopharmaceutical drug therapies currently on the market, with more than 100 approved and over 1000 more currently in clinical trials.^1,2^ Many of these recombinant proteins are produced using Chinese hamster ovary (CHO) cell expression systems. During the cell culture phase of production, endogenous proteins from these expression systems, referred to as host cell proteins (HCPs) are also generated. HCPs have the potential to be detrimental to both product efficacy and patient safety as some have been found to compromise product stability while others have biological activity within patients, including the potential to induce immunogenic responses.^3–6^ For that reason, HCPs are categorized as critical quality attributes (CQAs) that need to be monitored.

Despite HCPs being considered as CQAs, there is no specific threshold for a universally accepted aggregate level of residual HCPs present on a final drug product. While an informal target of below 100 ng HCPs/mg of product (or 100 ppm) has been a historical benchmark,^7,8^ this limit is inadequate for assessing risks associated with HCPs.^9,10^ Therefore, quality management and regulatory frameworks have been put in place to manage product quality and patient safety. Guidance for testing HCPs state that HCPs need to be managed to acceptable levels (The International Council for Harmonization (ICH); ICH-Q6B^11^). Since HCP composition and concentration is dependent on the host cell line and manufacturing process conditions, products are reviewed on a case-by-case basis, evaluating a multitude of HCP risk factors including identity of the HCPs (and their similarities to their human counterparts if the host cell line is nonhuman), prior experience with the therapeutic (i.e., how early in the development process an HCP was detected), the patient population, the therapeutic mechanism of action (i.e., does the therapeutic modulate the immune system), the route of administration, the exposure duration and the dosage frequency.^12,4^

Multianalyte enzyme-linked immunosorbent assays (ELISAs) are the current gold standard for determining total HCP concentration and are the primary release assay used in industry.^10^ However, they are not without limitations. Non- and weakly immunoreactive HCPs will not be detected and information on individual HCPs cannot be obtained.^8^ Therefore, a variety of orthogonal HCP monitoring strategies have been developed.^6^ Liquid chromatography–mass spectrometry (LC-MS) has become a widely used approach for orthogonal HCP analysis as LC-MS workflows, in particular bottom-up proteomics workflows, are capable of providing information on many individual HCPs simultaneously.^13,14^ For a single or limited number of HCPs, multiple reaction monitoring (MRM) and parallel reaction monitoring (PRM) can provide robust and sensitive HCP quantitation.^15–18^ However, for untargeted HCP quantitation, a major limitation of LC-MS strategies is the ability to detect low-level HCPs in the presence of the protein biotherapeutic. This can be due to a variety of factors including the broad dynamic range between the protein biotherapeutic and the HCPs and the potential for product peptides to interfere with the detection of HCP peptides due to coelution or similar mass-to-charge ratios (*m*/*z*) which can cause overlapping isotope peaks. A variety of sample preparation strategies including affinity depletion and prefractionation,^15,19–24^ the use of molecular weight cutoff filters,^25^ and alternative digestion strategies to bottom-up proteomics, such as native digestion^26–29^ have been successfully utilized to reduce sample dynamic range and product peptide interference for protein biotherapeutic HCP analysis.

Furthermore, advancements in MS instrumentation such as the development of analysers capable of high resolution-accurate mass spectrometry (HRAMS) have improved MS1 and MS2 (MS/MS) acquisition capabilities. Acquisition strategies using HRAMS such as high-field asymmetric waveform ion mobility spectrometry (FAIMS),^29,30^ sequential window acquisition of all theoretical spectra (SWATH),^31–33^ and the “Boxcar” MS acquisition method^34,35^ have enhanced the dynamic range and peptide detection capabilities of MS instrumentation, thus improving HCP detection. Additionally, HRAMS has improved the data-independent acquisition (DIA) capabilities of MS instrumentation, which can help expand the dynamic range of an MS instrument, compared to data-dependent acquisition (DDA), which many of the HCP analysis strategies utilize. DDA only fragments a preselected number of the most abundant peptides while DIA analysis fragments all peptides regardless of abundance. This results in a more comprehensive HCP analysis including improved detection of low abundant HCPs.^36–39^

The recently developed Thermo Scientific Orbitrap Astral mass spectrometer can further improve protein biotherapeutic HCP detection and monitoring capabilities. The Orbitrap Astral MS is a HRAMS hybrid instrument which combines a mass-resolving quadrupole, an Orbitrap mass analyzer, and the novel asymmetric track lossless (Astral) analyzer, to provide faster acquisition of high-resolution MS/MS spectra than standard state-of-the-art mass spectrometers, high dynamic range and resolution, as well as high sensitivity.^40^ Furthermore, the speed and sensitivity of the Orbitrap Astral MS allows for the use of shorter separation gradients without the loss of protein identifications, helping to facilitate high-throughput sample analysis.^40–42^ Proteomic profiling and quantitative proteomics analysis of complex sample matrices using the Orbitrap Astral MS have demonstrated significant improvements in analysis times, protein identifications, or both, compared to previously utilized methods and instrumentation.^41–45^ Additionally, a recently presented benchmarking study of the Orbitrap Astral MS for HCP analysis using NISTmAb spiked with CHO HCPs describes how the Orbitrap Astral MS supports high-fidelity HCP quantitation with DIA outperforming DDA, illustrating its potential to characterize HCPs on a broad spectrum of protein biotherapeutics.^46^

In this work, we demonstrate how the Orbitrap Astral MS can be utilized to monitor HCPs on commercially available protein biotherapeutics without the need for complex sample preparation strategies. The speed and sensitivity of the instrument enabled utilization of a 60 samples-per-day (SPD; 24 min, injection-to-injection) LC method for high-speed MS^2^ analysis. A total of 36 protein biotherapeutic drug product batches from 21 products were tested. This cohort was comprised of IgG1, IgG2, IgG4, bispecific and Fc-fusion proteins enabling us to survey the HCP populations across drug products and batches.

## Experimental Procedures

### Experimental Design and Statistical Rationale

Triplicate digestions were performed on each protein biotherapeutic product analyzed (36 product samples in total). After digestion a known quantity of predigested Hi3 *Escherichia coli* peptide internal standard was spiked into the samples for subsequent HCP quantitation.^47^ Each digestion was analyzed using LC-MS/MS with duplicate technical injections. For each product analyzed the 6 samples (digestion triplicates × duplicate technical injections) were processed together during data processing. The false discovery rate (FDR) at the peptide spectrum match (PSM), peptide, and protein level was set to 1%. A protein was considered identified if ≥2 unique peptides were assigned to the protein in both injections of at least one of the triplicate digestions. This was chosen to assess the ability of the Orbitrap Astral MS to detect low level HCPs even in the presence of many other HCPs. HCP quantitation was assessed relative to the predigested *E. coli* peptides spiked-in to the samples before LC-MS/MS analysis, an approach which follows the guidance of the U.S. Pharmacopeia (USP<1132.1>2023) as discussed in Chrone et al.^14^ This Hi3 quantitation approach determines HCP concentrations relative to the summed signal intensities of the three most abundant peptides of the *E. coli* peptides.^47^

### Samples

A variety of protein biotherapeutic aliquots including abatacept (2 batches), adalimumab (2 batches), aflibercept (4 batches), alemtuzumab (1 batch), amivantamab (1 batch), bevacizumab (2 batches), denosumab (1 batch), emicizumab (1 batch), etanercept (3 batches), ipilimumab (1 batch), ixekizumab (1 batch), luspatercept (1 batch), nivolumab (2 batches), obinutuzumab (1 batch), panitumumab (1 batch), pembrolizumab (1 batch), rituximab (2 batches), secukinumab (1 batch), tocilizumab (1 batch), trastuzumab (6 batches), and vedolizumab (1 batch) were either provided by collaborators or commercially sourced from Evidentec GmbH (Potsdam, Germany) (Table S1).

### NISTmAb Sample Preparation for Benchmarking of Orbitrap Astral MS Performance

To illustrate the capabilities of the Orbitrap Astral MS, we analyzed NISTmAb prepared using native digestion to compare results against literature using similar sample preparation strategies. NISTmAb reference material standard RM8671 was purchased from Millipore Sigma (Merck). Native digest of the NISTmAb sample was performed in duplicate following a slightly modified version of the procedure outlined in Beaumal et al.,^39^ which itself was adapted from the protocol developed by Huang et al.^26^ Briefly, for each digest, 1 mg (100 μL of 10 mg/mL mAb) was supplemented with 90 μL of ultrapurified water and 5 μL of 1 M Tris-HCl, pH 8. Tryptic digestion was performed overnight at 37 °C using a 1:400 trypsin/protein ratio. After digestion, 1.5 μL of 303 mM dithiothreitol (DTT; Sigma) were added with subsequent incubation for 10 min at 90 °C to perform disulfide bond reduction. Centrifugation at 13,000 × g for 2 min was performed to pellet the reduced mAb and the supernatant was transferred to a fresh tube where it was acidified with 0.5 μL of formic acid (FA). Samples were subsequently dried down using a speed-vac until ready for analysis. Prior to LC-MS analysis, samples were reconstituted in 2% acetonitrile (ACN) and 0.1% FA to obtain a theoretical final concentration of 1 μg/μL based on the original amount of starting material. Based on this theoretical concentration, 500 ng of sample was loaded onto the column during LC-MS/MS analysis during each injection.

SP3 protocol was performed on NISTmAb to benchmark our approach against native digestion of protein therapeutics. SP3 protocol was performed in duplicate following the procedure described in the HCP Sample Preparation section.

### HCP Sample Preparation

All protein biotherapeutics were digested using a modified version of the single-pot solid-phase-enhanced sample preparation (SP3) protocol described by Hughes et al.^48^ and previously utilized in Strasser et al.^37^ For each protein biotherapeutic, 50 μg of protein biotherapeutic was brought to a final volume of 95 μL with 50 mM ammonium bicarbonate, pH 8.0 (prepared from BioUltra, ≥99.5% grade; Sigma). Reduction was performed using freshly prepared DTT (2.5 μL) at 56 °C for 20 min, followed by alkylation with freshly prepared iodoacetamide (IAA; Sigma; 2.5 μL) for 15 min at room temperature in the dark. A final concentration of 0.2 M DTT and 0.4 M IAA were used, respectively. Samples were then bound to a 1:1 mixture of hydrophilic (5 μL) and hydrophobic (5 μL) Sera-Mag SpeedBeads Carboxyl Magnetic Beads (Cytiva) and washed with 80% ethanol using a KingFisher Duo Prime purification system under the control of Thermo Scientific BindIt software, version 4.0 (Thermo Fisher Scientific). Samples still bound to the beads were then transferred to clean low-bind 1.5 mL tubes and digested with 1 μg of trypsin (Thermo Scientific MS grade, Fisher Scientific) for 4 h at 37 °C shaking at 700 rpm. The digestion was subsequently quenched with trifluoroacetic acid (final concentration 0.1%). Samples were then centrifuged for 5 min at 14,000 rpm to pellet any residual magnetic beads before being transferred to clean low-bind 1.5 mL tubes. Finally, samples were dried down in a speed-vac and stored at −20 °C until ready for LC-MS analysis. Triplicate digestions were performed for each sample with all digestions for a sample performed together with the goal of minimizing variability between the digests.

### LC-MS Analysis

For LC-MS analysis, each digested sample was reconstituted in 50 μL of 0.1% FA in water. Five μg (5 μL) of sample was transferred to a 0.2 mL SureSTART screw vial (Thermo Fisher Scientific) where 500 fmol of Hi3 *E. coli* peptide standard (Waters), previously reconstituted in 0.1% FA in water, were added. The sample in the vial was then brought to a final volume of 50 μL using 0.1% FA in water. All 0.1% formic acid in water used in the preceding steps described was Optima LC-MS grade purchased from Fisher Scientific.

For each protein biotherapeutic digest, duplicate 500 ng injections were performed, totalling 6 injections per protein biotherapeutic product. After these 6 injections a solvent-only blank was run to ensure no carry over between each biotherapeutic analyzed. All analyses were performed using a Thermo Scientific Vanquish Neo UHPLC system, operated in Trap & Elute mode, coupled to an Orbitrap Astral MS with a Thermo Scientific Easy-spray source, operated in data-independent acquisition (DIA) mode (Thermo Fisher Scientific, Bremen, Germany). Samples were loaded onto a PepMap Neo Trap Cartridge (5 μm, 100 Å, 300 μm × 5 mm; Thermo Fisher Scientific) before separations were performed on an EASY-Spray PepMap RSLC C18 column (2 μm, 100 Å, 150 μm × 15 cm; Thermo Fisher Scientific), heated to 50 °C. A 60 SPD method operating at a flow rate of 800 nL/min was used for sample separations. This method includes a 19.9 min two-part gradient from 8 to 35% B (an 8–22.5% B gradient over 13 min immediately followed by a 22.5–35% B gradient over 6.9 min). Full separation method details are described in Table S2. Mobile phase A was 0.1% FA in water and mobile phase B was 0.1% FA in 80% acetonitrile. Both mobile phase A and B were Optima LC-MS grade purchased from Fisher Scientific.

DIA-MS analysis was performed in positive ion mode using a spray voltage of 1,900 V, an ion transfer tube temperature set to 290 °C, and an expected peak width of 10 s. Peptide application mode with standard pressure mode settings were used. MS1 scans were acquired in the Orbitrap at a resolution setting of 240,000 (@*m*/*z* 200) every 0.6 s across a scan range of 380–980 *m*/*z*. The maximum injection time was 5 ms and the number of microscans was 1. The normalized AGC target and RF lens settings were 500% and 40% respectively. MS/MS (MS2) spectra were acquired in the Astral analyzer across a scan range of 150–2,000 *m*/*z*, where the precursor mass range was set the same as the MS1 scan range. The isolation window and maximum injection time used were *m*/*z* 4.9 and 10 ms, respectively. The normalized HCD collision energy was set to 25%. The number of microscans, normalized AGC target, and RF lens settings were the same as those used for MS1 scans. Full method details are described in Table S3.

### Data Processing

Raw DIA data were processed with Thermo Scientific Proteome Discoverer software version 3.1 (from now on referred to as PD 3.1), utilizing the CHIMERYS intelligent search algorithm^49^ and INFERYS 3.0.0 fragment intensity prediction model.^50^ Trypsin/P was with full enzymatic specificity was used as the proteolytic enzyme and search criteria allowed a maximum of two missed cleavages, a peptide length of 7–30 residues, a peptide charge state range of 1–4, and a fragment ion mass tolerance of 10 ppm. Carbamidomethylation of cysteines was applied as a static modification while oxidation of methionine was set as a variable modification. To help with data processing due to the complexity of DIA data, deamidation was not included as a variable modification. The FDR for PSMs during processing was 1% while peptides and proteins were validated using a confidence threshold target FDR of 1% as well. For each protein biotherapeutic, the data files generated during the analysis of the triplicate digests (2 injections per digest for 6 data files in total) were processed together using the “One Job for All Files” processing mode. The search databases comprised of the respective protein biotherapeutic sequences, the *Cricetulus griseus* (CHO) Uniprot reference proteome database (Downloaded August 22, 2024), and the sequence of the Hi3 *E. coli* standard (provided by Waters). Additionally, the common Repository of Adventitious Proteins (cRAP) and MaxQuant contaminant databases were searched and utilized to identify and filter out potential protein-based contaminants introduced during sample preparation (from now on referred to as sample preparation protein contaminants or SPPCs). Finally, it needs to be mentioned that during publication of this manuscript, UniProt was in the process of undergoing a significant transition, affecting many proteomes including CHO. All MS proteomics data is available on the PRIDE partner repository with the data set identifier PXD067004.

### HCP Identification and Quantification

Protein identification and quantification was performed based on the validation filters described in Pythoud et al.^38^ and Beaumal et al.,^39^ but with some slight modifications. Briefly, for each protein biotherapeutic analyzed in PD 3.1, the peptides identified during data processing were filtered to remove peptides related to SPPCs as well as any keratins not already contained within the SPPC database. The remaining peptides were exported to an Excel file and then further filtered to remove any peptides shared between multiple proteins. A unique peptide was only kept if it was found in both injections of at least one sample digest. All peptides including the modified peptides searched were kept if they met the above criteria. Finally, a BLAST search of the identified peptides against the protein biotherapeutic sequence of the sample analyzed and the contaminant sequences was performed to determine if they might originate from the protein biotherapeutic due to sample degradation or unspecified product cleavage. Peptides longer than 5 amino acids, found to have a 100% identity and greater than 80% query coverage were removed. After filtering, a minimum of two unique peptides were required to consider a protein identification valid. Protein quantification was performed using the Top3 strategy described by Silva et al., which requires a minimum of 3 unique peptides for quantitation.^47^ Quantifiable protein concentrations were calculated using the known concentration of the Hi3 *E. coli* peptide internal standard spiked into each sample along with the molecular weight of the HCPs and the amount of protein biotherapeutic injected. All HCP concentrations are based on the summed signal intensities of the three most abundant HCP peptides relative to the intensities of the spiked in peptides. These were determined at the fragment-ion level using the area of the peptide spectral matches for each MS/MS spectra as determined during processing with PD 3.1. This process does presuppose that the response factor for the HCP peptides and the Hi3 *E. coli* peptides is the same, though there is potential this is not the case especially for very low abundant HCPs that might be masked by more prominent peptides. Calculated concentrations are described in ng of HCP/mg of product. Result of this data processing step can be found in Table S4.

### GO Term Enrichment

GO term enrichment of the identified HCPs was performed in DAVID^51,52^ to see what molecular functions (MF) they were associated with. The CHO background in DAVID was used. The GOTERM_MF_ALL Chart was selected to identify which MF was associated with the most HCPs. An Ease Score of 0.01 and a count of 3 was used to filter the results. The Benjamini option was selected to determine enrichment significance. Only MFs with Benjamini–Hochberg (B–H) adjusted *p*-values ≤ 0.01 were considered to be significantly enriched. To identify the direct MFs associated with the identified HCPs, the GOTERM_MF_DIRECT Chart was selected. The same filter parameters as used with GOTERM_MF_ALL were applied. REVIGO was subsequently used to visualize relationships between the direct MF GO terms.^53^ The interactive graph was utilized to illustrate the GO term relationships with the color of the bubble corresponding to the input B–H adjusted *p*-value (lighter color corresponds with smaller *p*-value) while the size of the bubble corresponds to the LogSize value of the GO term. Where GO terms have high degrees of similarity their edges are linked with gray lines, where line thickness corresponds with degree of similarity (thicker line equates to higher similarity). The tree map in REVIGO illustrates high-level groupings of the input GO Terms, shown as rectangles. Rectangle size corresponds to level of enrichment, with larger squares illustrating greater enrichment (i.e., smaller *p*-values). Similar groups are clustered together and color coded.

### HCP Interaction Mapping

Mapping of the potential interactions between the identified HCPs was performed using STRING database v12.0.^54^ A list of the HCP identifications was loaded into STRING and searched against the CHO organism database. Only interactions with high confidence (0.7 or greater) and a high FDR stringency (1%) were mapped with each node representing an HCP known to be part of a physical or functional network. Nodes are connected by edges (solid lines) whose thickness indicates the strength of the support behind the interaction. The MCL option in STRING, which utilizes the Markov Cluster (MCL) algorithm to provide scalable unsupervised clustering in graphs based on the simulation of graphical stochastic flow,^55^ was used to illustrate the natural clusters within the data. The inflation parameter was set to 3 (default) and interactions between clusters was indicated using a dotted line.

### Identification of Potential High-Risk HCPs

Initial identification of potential high-risk HCPs was performed by consulting the publicly available list of high-risk HCPs determined by the Biophorum Development Group (BPDG), a cross-industry collaboration established in part to assist the industry in mitigating risks caused by the presence of potentially high-risk HCPs (Table S5 and https://www.biophorum.com/host-cell-proteins/, accessed September 10, 2024).^4,56^ However, this list is not currently maintained as the up-to-date version is behind a pay wall. To assess if any of the other HCPs could potentially be considered high-risk, we searched DAVID for genes functionally related to those considered high risk by the BPDG. Here the list of BPDG high-risk HCPs was uploaded to DAVID as a gene list. Then for each HCP we searched for functionally related genes in CHO with a similarity score (Kappa) ≥0.5 (scale 0–1) and an overlap ≥3. If any of the HCPs identified during our analysis matched these criteria when searching the list of BPDG high-risk HCPs, they were considered to be potentially high risk, but for the purposes of this manuscript, are referred to as potentially problematic HCPs. Additionally, we included potential smuggling HCPs, such as histones, as potentially problematic, due to their potential to help carry more harmful HCPs through the downstream purification process.^57^

## Results and Discussion

### Benchmarking Orbitrap Astral MS Performance Using NISTmAb

NISTmAb reference material was initially analyzed to evaluate the performance of the platform. Two sample preparation strategies were employed: native digest as described by Huang et al.^26^ and the SP3 protocol employed throughout this study. Duplicate digests and technical injection triplicates of each digest were analyzed for each sample preparation strategy. Data analysis allowed us to identify 124 HCPs using the native digest strategy. We compared these identified HCPs with those HCPs identified in studies applying similar digest strategies based on the Huang et al. sample preparation method^18,21,26,39,58^ (Figure 1A). Only the study by Beaumal et al.^39^ identified more HCPs, but the difference in total identifications was small with only 9 additional HCPs detected in their study. However, a key benefit of our study is that the speed and sensitivity of the Astral mass analyzer facilitate a reduction in LC separation gradients. Our results were achieved utilizing a 60 SPD (24 min) LC separation method (19.9 min two-part gradient) compared to the 2 h (12 SPD) method employed by Beaumal et al.^39^ Standard LC run times for comparable HCP studies ranged from 44 min to 2 h (12 SPD to 30 SPD) to enable sufficient HCP identification.^6,21,58–60^ Ma et al.^21^ employed the fastest LC method (44 min), but only detected 80 HCPs in their study. The fact that we achieved similar total HCP identification numbers as Beaumal et al. while using a separations method shorter than the one employed by Ma et al. illustrates the potential benefits using the Orbitrap Astral MS can have when applied to HCP analysis.

**Figure 1:**
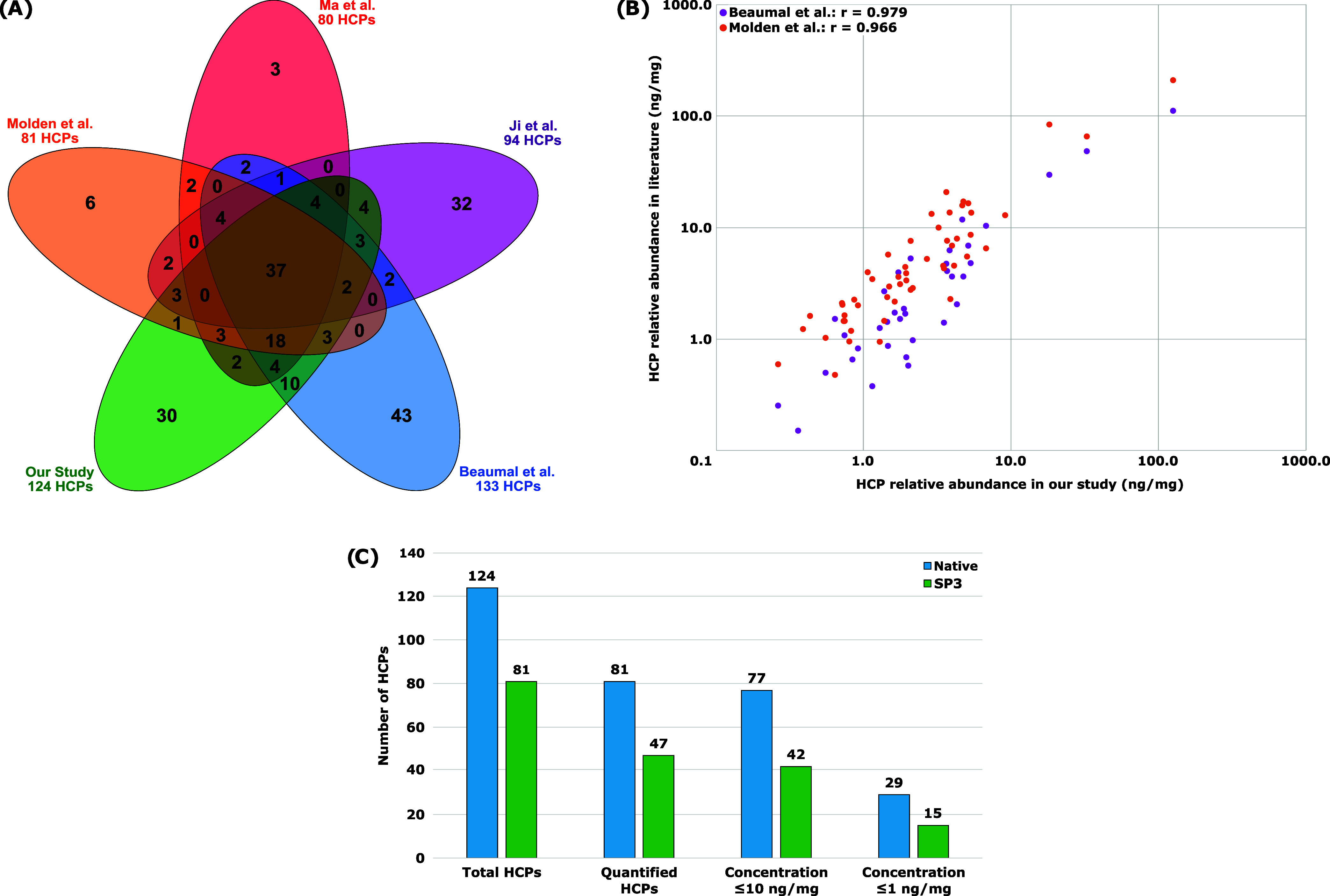
Benchmarking of instrument performance using NISTmAb. (A) Venn diagram comparing HCPs identified on NISTmAb in Molden et al.^58^ (Orange), Ma et al. (44 min)^21^ (Red), Ji et al.^18^ (Purple), Beaumal et al.^39^ (Blue), and our study (Green). For all studies native digestion of NIST was performed based on the procedure described in Huang et al.^26^ Data from all studies was acquired using nanoLC-MS/MS with the exception of Molden et al. and Ji et al., whose studies utilized ultra performance LC-MS/MS. (B) Pearson coefficient and correlation between the individual amounts of HCPs in ng of HCP per mg of NISTmAb in our study versus previously reported quantities obtained by Beaumal et al. (Purple) and Molden et al. (Orange). Concentrations for Molden et al. were converted from the micromoles to moles ratio reported in their study to ng of HCP to mg of mAb for standardization purposes. (C) Comparison of HCPs identified using the native digestion method and the modified SP3 protocol described by Hughes et al.^48^ 1 mg of mAb was digested for the native digest while only 50 μg of mAb was digested for the SP3 protocol.

Overall, considerable overlap was seen between our study and the others examined with 61 (49%) of our identified HCPs found in at least three other studies including 37 (30%) HCPs identified in all studies compared (Figure 1A). Furthermore, an additional 33 HCPs (27%) were identified in at least one other study. We also identified 30 HCPs not detected in the other studies. The number of HCPs quantified with a minimum of 3 unique peptides using Hi3 quantitation in our study was 81 (65%). Many of these HCPs were identified at low or trace-levels with 77 (95%) HCPs having concentrations below 10 ng/mg including 29 (36%) HCPs with sub 1 ng/mg concentrations. Of those quantified HCPs, 51 (63%) were identified in at least three other studies including 35 (43%) HCPs identified in all studies compared (Figure S1). Furthermore, an additional 17 quantifiable HCPs (21%) were identified in at least one other study. Overall, 84% (68 HCPs) of all quantified HCPs identified were also identified in at least one other study.

We next evaluated the quantification comparability of our results against published data, specifically the findings in Molden et al.^58^ and Beaumal et al.,^39^ as shown in Figure 1B. Here it should be reiterated that quantitation was performed using spiked-in peptides assumed to have a similar response time as the HCP peptides, since the HCPs were unknown before analysis. This approach follows the guidance of the U.S. Pharmacopeia (USP<1132.1>2023) as discussed in Chrone et al.^14^ Comparing common HCPs, we saw a strong correlation in the quantities calculated except for the HCP fructose-bisphosphate aldolase A (P05064). The Pearson coefficient for the 36 HCPs in common between our study and the Beaumal et al. study was 0.67 when P05064 was included, but 0.98 when P05064 was excluded. Similarly, the Pearson coefficient for the 54 HCPs in common between our study and the Molden et al. study was 0.79 when P05064 was included, but 0.97 when P05064 was excluded.

Along with benchmarking the performance of the Orbitrap Astral MS against previous studies, we also compared the native digest strategy with a modified version of the SP3 digestion strategy developed by Hughes et al.^48^ (Figure 1C). Here the SP3 protocol identified 81 HCPs compared to the 124 HCPs detected using native digest sample preparation. This is most likely because a significantly smaller amount of mAb is being digested with the SP3 method (50 μg) than with the native method (1 mg), which can influence the number of HCPs detected even when the same amount of sample is analyzed with LC-MS. Furthermore, only 29 of the identified HCPs were common across both digestion strategies Figure S2. This is in part due to the lesser amount digested, but it also illustrates how different sample preparation strategies can influence which HCPs are detected, as suggested by Ji et al.^18^ Of the identified HCPs in the SP3 protocol, 47 (58%) were quantified with a minimum of 3 unique peptides using Hi3 quantitation. As with the native digest, many of these HCPs were identified at low or trace-levels with 42 (89%) HCPs having concentrations below 10 ng/mg including 15 (32%) HCPs with sub 1 ng/mg concentrations. As a focus of this study is high-throughput from sample preparation through analysis, the SP3 digestion method was utilized in this study because of its relatively short and partially automated sample preparation (4 h digestion time compared to overnight digestion with the native protocol). This helped facilitate the increase in throughput for this large cohort of samples consisting of 36 protein biotherapeutic samples each digested in triplicate.

### HCP Identification across Commercial Biotherapeutics

In this study, thirty-six protein biotherapeutics including multiple batches of the same product in some cases, were analyzed. This included 18 IgG1, 2 IgG2, 4 IgG4, 2 bispecific, and 10 Fc-fusion products. We initially explored all HCPs identified across the analyzed products. Upon examination of the detected HCPs, we noticed the presence of a set of CHO Ig-like proteins predominantly found to have only 2 or 3 unique peptides associated with them (Table S6). Their abundance (or estimated abundance when only identified with 2 unique peptides) was well above 100 ng/mg. As such, we suspected that these identifications might originate from the protein biotherapeutic products themselves, possibly due to product degradation, and are not actually real HCPs. While it is beyond the scope of this paper to confirm this experimentally, we reviewed the literature where native digestion for CHO based protein biotherapeutic HCP analysis was performed to see if such proteins were detected. As these procedures are used to remove the protein biotherapeutic during sample preparation, if such proteins are identified in their data, that would suggest that the Ig-like proteins are actual HCPs instead of parts of the protein biotherapeutic. However, none of the Ig-like proteins were identified in those papers, and so it was considered that the proteins were actually originating from the protein biotherapeutic instead of true HCPs. Thus, for the purposes of this study, they were excluded from our results.

Furthermore, as an additional precaution against identifying contaminant proteins as HCPs, we searched the gene ontology (GO) of our identified proteins in Uniprot for proteins found in the desmosome cellular component or that are involved in the biological process of keratinization. This is because proteins found in the desmosome and related to keratinization are highly abundant within skin cells^61,62^ and as such their presence is likely due to sample handling/processing contamination. To this end, seven proteins were removed from our results (Table S7).

After filtering, a total of 236 individual HCPs were identified with a unique Uniprot accession number (Table S8). Some identifications include instances where proteins with the same protein name are assigned unique accession numbers in Uniprot because they are associated with different genes. Of these, 129 HCPs (55%) were found to be quantifiable with 3 unique peptides in at least one therapeutic using Hi3 quantitation. Using the molar concentrations of the quantifiable HCPs detected and their respective protein biotherapeutics, we determined that quantifiable HCPs were detected at concentrations between to 3–5 orders of magnitude lower than their associated protein biotherapeutics, with HCPs quantifiable at attomole concentrations.

Plotting the ranked abundance of the quantifiable HCPs detected across all the protein biotherapeutics illustrates the wide range of concentrations HCPs were detected at, including at sub 1 ng/mg amounts (Figure 2). In total, there were 422 instances where an identified HCP could be quantified (Table S9). The most abundant HCP detected in any specific product was signal-regulatory protein β-2-like found in panitumumab at a concentration of 336.99 ng/mg, while the least abundant protein detected was LSM6 homologue found in etanercept batch 2 at a concentration of 0.2 ng/mg. A great majority of the quantifiable HCPs were detected at low individual concentrations with 93% (392 HCPs), 79% (333 HCPs), and 8% (35 HCPs) detected at concentrations below 20 ng/mg, 10 ng/mg and 1 ng/mg, respectively. Conversely, 2 HCPs, the aforementioned signal-regulatory protein β-2-like protein and HtrA serine peptidase 1 (134.6 ng/mg, ixekizumab) were detected at individual concentrations greater than 100 ng/mg. Finally, variance in HCP concentrations was observed across all quantifiable HCPs, though HCPs with higher concentrations generally showed greater variance than HCPs found at lower concentrations (Table S9).

**Figure 2:**
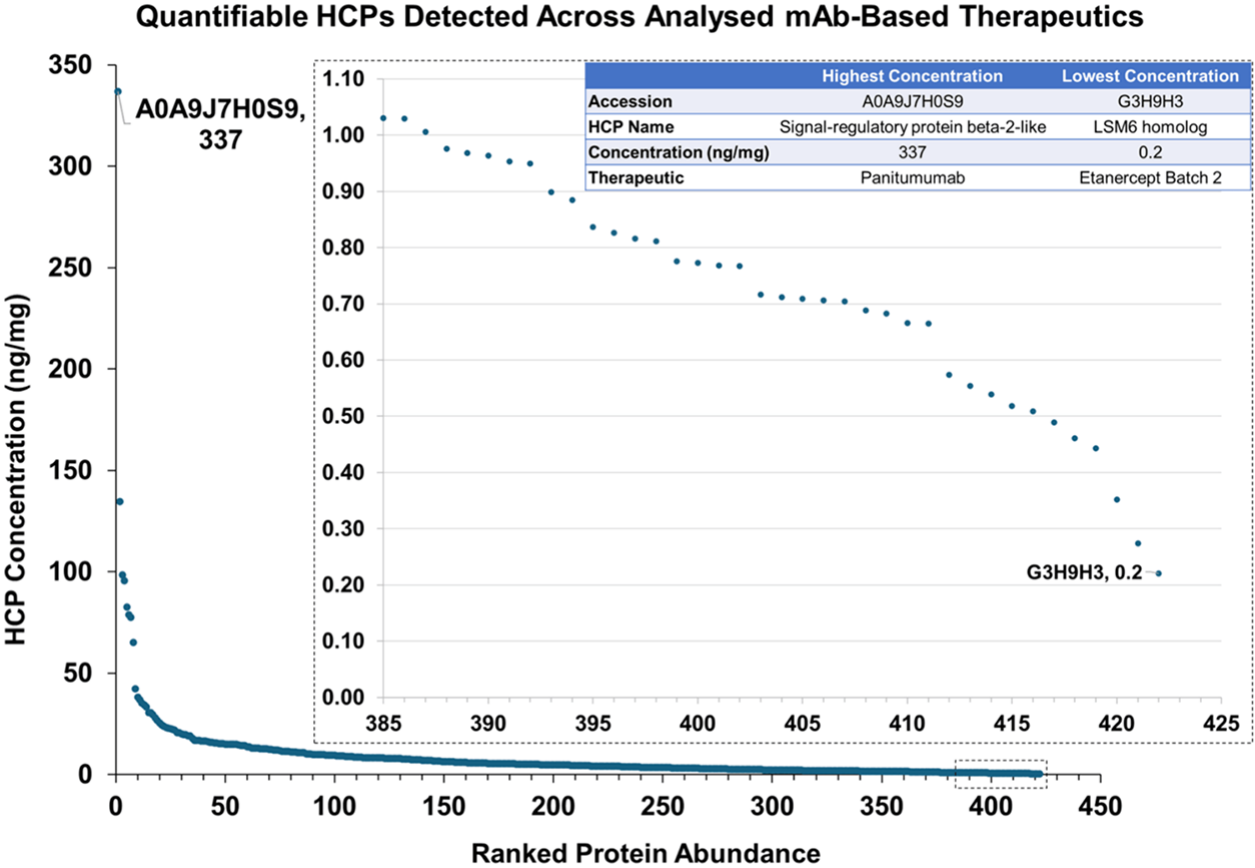
Ranked abundance plot of all times HCPs were found to be quantifiable. Hi3 quantitation, which requires a minimum of 3 unique peptides to be detected, was used for quantitation. Insert (dashed black box) highlights all times HCPs were quantified at a concentration below 1 ng/mg. Table in upper right corner details the most abundant and least abundant HCP detected across all HCPs analyzed products. For clarities sake standard deviation error bars were left out of this figure. Standard deviations and coefficient of variation values for these HCPs can be found in Table S9.

Ranking the detected HCPs by frequency of identification and the drug products by number of proteins identified was performed to determine the HCP profiles of each therapeutic (Figure S3). Here, where proteins were only detected with 2 unique peptides, their concentrations were estimated using those two peptides only. The generated heat map shows great heterogeneity in the HCP profiles of each drug product. Most ubiquitous were actin, cytoplasmic proteins 1 and 2 (present in 27 products combined), glyceraldehyde-3-phosphate dehydrogenase proteins (23 products combined), histone H4 proteins (22 products combined), annexin proteins (23 products combined), elongation factor 1-α proteins (21 products combined) and the 14–3–3 protein zeta/delta (18 products), all found to be present at least 50% of the products analyzed. Additionally, 23 HCPs had an incidence rate greater than 20%. Conversely, 75% of the identified HCPs were detected in 3 or fewer therapeutics.

### HCP Identification in Individual Products

From the results of our study, thirty-two (89%) of the 36 products analyzed contained fewer than 40 HCPs with 50% (18) of all products containing 10 or fewer HCPs (Figure 3A). The average number of HCPs per product identified and quantified was approximately 20 and 12 HCPs respectively (dotted vertical line in Figure 3B). The broad nature of the HCP distribution across the products analyzed is most likely due to a variety of reasons including the product type and the downstream purification processes utilized during production. For instance, Fc-fusion products are often more sensitive to the low-pH conditions utilized during Protein A purification than standard mAb products, requiring the development of various strategies to minimize the presence of Fc-fusion product aggregation during protein A purification.^63^ It is also known that different HCPs can have varied affinities to a drug product type, causing them to bind and coelute with the product during purification.^13,58,64–68^ Histones, known to contribute to HCP smuggling during purification,^57,63^ were one of the protein types observed widely across the products analyzed and might also contribute to larger numbers of HCPs in certain products.

**Figure 3:**
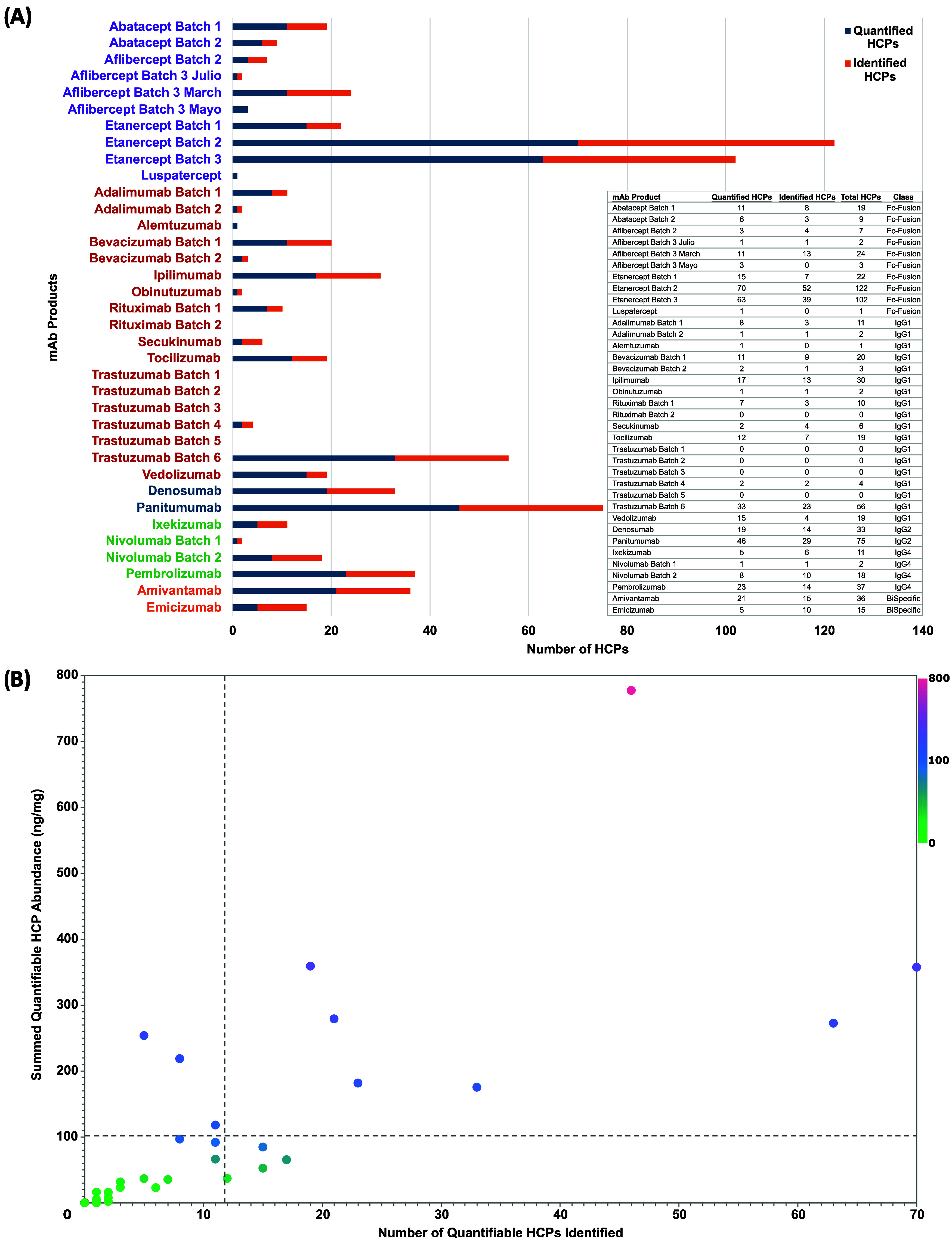
HCP profiles of Protein biotherapeutics. (A) Horizontal bar graph showing the identified and quantified HCPs detected on each protein biotherapeutic analyzed. Dark blue portion of each bar represents the number of HCPs with ≥3 unique peptides allowing for Hi3 quantitation, while the orange portion represents the additional HCPs identified, but with only 2 unique peptides. Table in lower right corner lists the total number of HCPs detected per therapeutic. Product names on *y*-axis are ordered and colored by class: Fc-fusion–Purple, IgG1–Red, IgG2–Blue, IgG4–Green, Bispecific–Orange. (B) Quantified HCP numbers in each product compared to their aggregate concentration. HCP concentrations were determined using Hi3 quantitation. Dotted vertical line: Average number of HCPs found per product. Dotted horizontal line: Average aggregate concentration of quantifiable HCPs per product.

An estimation of the total HCP content within the protein biotherapeutics was performed by summing the concentrations of their respective quantifiable HCPs. The respective summed quantifiable HCP concentrations were then mapped against the total number of quantifiable HCPs in their respective protein biotherapeutics (Figure 3B). In general, higher numbers of quantifiable HCPs detected corresponded with higher summed HCP concentrations. The average total summed product HCP concentration was 102.7 ng/mg (dotted horizontal line in Figure 3). However, 72% (26) of the protein biotherapeutics analyzed had total HCP concentrations lower than 100 ng/mg. Those with HCP concentrations greater than 100 ng/mg generally contained the most quantifiable HCPs, following the observed trend correlating increased HCP numbers with increased total HCP concentrations. However, in a few instances, the high total concentration was influenced by the high concentration of 1 or 2 individual HCPs.

Throughout this study a 100 ng of HCP/mg of product threshold has been applied as the maximum total amount of HCP impurities considered allowable on a product. However, it must be acknowledged that while this is a historically used target,^69,70^ advances in manufacturing processes and improved understanding of HCPs in general have rendered this approach insufficient. Now this target is an oft used rule of thumb for benchmarking manufacturing capabilities but is not regulatory guidance.^4,9^ Current guidance does not have a set limit but recommends achieving the lowest level of HCPs technically possible and evaluation of each product on a case-by-case basis (USP<1132.1>2023; ICH-Q6B).

It also must be mentioned this 100 ng/mg value is based on ELISA methods used to monitor HCP concentrations and that these concentration readings do not directly translate to concentrations determined using MS analysis. There are a variety potential issues with determining HCP concentrations solely using ELISA. They have been found to preferentially select immunogenic HCPs, often failing to detect nonimmunoreactive and weakly immunoreactive HCPs.^8^ Additionally, they rarely detect histones, having been found in some cases to underestimate their amount by more than 20,000-fold.^71^ They also can have low sensitivity and most importantly do not allow for individual HCP quantitation.^5,57,72^ As MS instrumentation becomes more powerful, highly sensitive instrumentation such as the Orbitrap Astral MS have the potential to detect and quantify individual HCPs at levels that further illustrate the inadequacy of this 100 ng/mg limit; at least when used to discuss HCP concentrations determined using MS analysis. In the meantime, the ability to detect high numbers of HCPs at such low concentrations can still be beneficial to understanding if specific types of HCPs are overly abundant on a product, facilitating the adjustment or establishment of purification strategies focused on minimizing their presence if required. Additionally, improved HCP detection enables better identification and quantitation of individual HCPs whose presence may be detrimental to the product efficacy or patient safety, even when present at low levels.

### HCP Comparison across Product Classes

An evaluation of HCPs identified across the different product classes was also performed. Here the HCPs found in all products were used. To do this we first grouped the products by their classifications resulting in five groups: Fc-fusion, IgG1, IgG2, IgG4, and bispecific. The HCPs identified on the products in each group were curated to generate a list of identified HCPs for each class. Here, we identified 173, 97, 83, 50, and 41 HCPs on the Fc-fusion, IgG1, IgG2, IgG4, and bispecific products, respectively. We also compared the HCPs found in each group to see how many proteins were unique to each classification (Figure 4). In this case 87, 30, 12, 12, and 5 HCPs were found to be unique to the Fc-fusion, IgG1, IgG2, IgG4, and bispecific products, respectively. Additionally, 19 HCPs were identified in all product classes. As discussed later, subsequent GO Term enrichment analysis showed that the molecular functions associated with the identified HCPs was varied across groupings, with some found to be unique to specific product classes.

**Figure 4:**
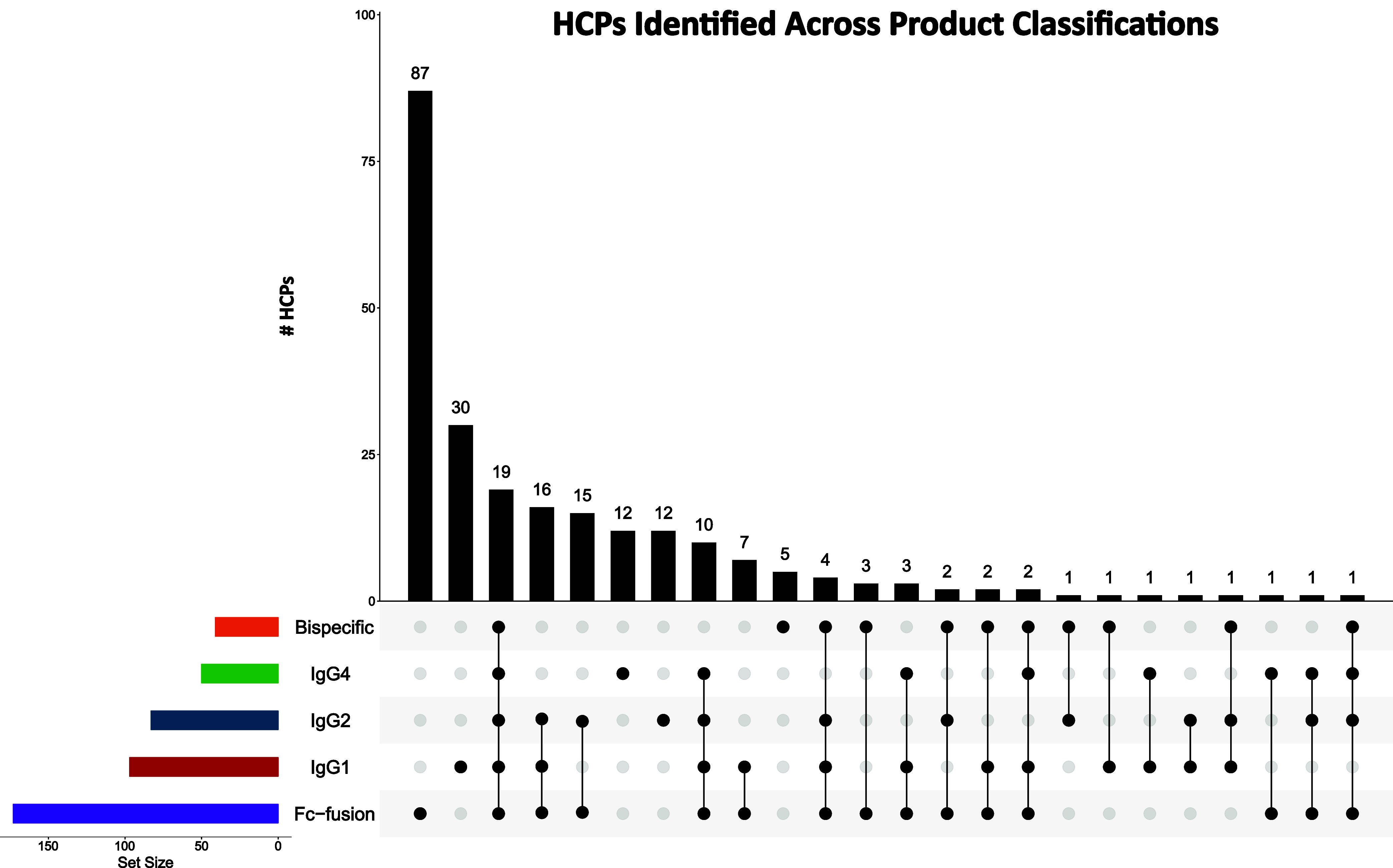
Upset plot comparing HCPs identified across product classifications. Identified HCPs were grouped according to the class of the product they were identified on, resulting in five HCP groupings: FC-fusion (purple), IgG1 (red), IgG2 (blue), IgG4 (green), and Bispecific (orange). A comparison was then performed to see what HCPs were similar and different across the five classes of protein biotherapeutics. The Upset plot was generated using the UpSetR Shiny App.^73^

### Physico-Chemical Properties of Identified HCPs

We explored the physicochemical parameters of identified HCPs to see if any commonalities could be identified across them. Using ProtParam (https://web.expasy.org/protparam/) we evaluated five standard protein physicochemical parameters: molecular weight (MW), isoelectric point (pI), protein stability, aliphatic index and hydrophobicity. This was performed by evaluating each property against the total number of identified HCPs (Figure S4). HCP protein sequences utilized for computation of their physicochemical parameters were obtained using their UniProt accession numbers. All calculations were based solely on the amino acid sequences, with no post-translational modifications potentially present on the HCPs considered. Analysis revealed that the most prominent characteristics of the HCPs identified were their hydrophobicity (Figure S4A) or their molecular weight (Figure S4B). Over 94% of the identified HCPs were found to be hydrophilic, as determined by having a GRAVY score of <0.^74^ Meanwhile over 89% of the detected HCPs had a MW below 100 kDa with 60% of them having a MW below 50 kDa. Another prominent feature of the identified HCPs was their potential thermal stability. The aliphatic index, which measures the relative volume of aliphatic side chains on a protein, is considered to be an indicator for protein thermal stability.^75^ A higher value generally corresponds to higher thermostability. In our findings (Figure S4C), 94% of HCPs had an aliphatic index greater than 60 and 64% had an index greater than 80, suggesting that many of the detected HCPs have some degree of thermal stability. Protein pI was more varied across the detected HCPs, with HCPs having a pI ranging from 4 to 12 and about 67% having a theoretical pI < 7 (Figure S4D). Protein stability was revealed to have minimal impact on the types of HCPs detected, with 51% found to be stable as measured by the instability index^76^ within Protparam (Figure S4E). We also charted the HCPs physicochemical properties against all instances where they were found to be quantified, to evaluate if there was any correlation between a distinct physicochemical property and an HCPs concentration (Figure S5). However, no correlation between protein concentration and physicochemical property was found.

### GO Term Enrichment for HCP Functionality

GO Term enrichment in DAVID was performed to evaluate the MFs associated with the HCPs identified across all the products analyzed (All_HCP). Here, 236 HCPs were mapped to 209 gene IDs associated with the CHO background in DAVID. Assessing all MF results (MF_ALL) revealed that the most gene IDs were associated with “catalytic activity” (46%, 97 gene IDs) and “protein binding” (44%, 91 gene IDs) (Table S10). Investigating the direct MF results (MF_DIRECT) showed that HCPs were associated with 13 terms, the most significantly enriched being “oxidoreductase activity, acting on the aldehyde or oxo group of donors, NAD or NADP as acceptor” with a B–H adjusted *p*-value of 7.20 × 10^–7^ (Tables 1 and S11). Using REVIGO we mapped the MF_DIRECT GO Terms to visualize the degree of similarities between them. GO Terms utilized can be found in Table S11.

**Table 1:** Cross Classification Comparison of Direct Molecular Function Enrichments in DAVID^a^,^c^,^d^,^e^,^f^,^g^

	All_HCP	Fc-fusion	IgG1	IgG2	IgG4
term	Benjamini–Hochberg adjusted *p*-value				
Oxidoreductase activity, acting on the aldehyde or oxo group of donors, NAD or NADP as acceptor	7.20 × 10^–07^	1.10 × 10^–07^	3.90 × 10^–09^	1.30 × 10^–09^	6.20 × 10^–11^
NAD binding	4.70 × 10^–06^	3.40 × 10^–07^	2.70 × 10^–05^	4.00 × 10^–07^	6.10 × 10^–07^
Actin filament binding	4.80 × 10^–06^	9.50 × 10^–04^	1.40 × 10^–03^	3.50 × 10^–03^	^b^
NADP binding	7.10 × 10^–06^	2.40 × 10^–05^	3.80 × 10^–08^	4.00 × 10^–07^	3.30 × 10^–10^
Structural constituent of cytoskeleton	1.50 × 10^–05^	2.60 × 10^–05^	2.10 × 10^–05^	4.00 × 10^–07^	7.40 × 10^–03^
Unfolded protein binding	7.60 × 10^–05^	7.10 × 10^–05^	3.10 × 10^–04^	^b^	^b^
Glyceraldehyde-3-phosphate dehydrogenase (NAD+) (phosphorylating) activity	1.10 × 10^–04^	2.40 × 10^–05^	2.10 × 10^–05^	4.40 × 10^–06^	3.80 × 10^–07^
ATP hydrolysis activity	4.70 × 10^–04^	1.10 × 10^–04^	^b^	^b^	^b^
ATP-dependent protein folding chaperone	5.10 × 10^–04^	1.10 × 10^–04^	2.00 × 10^–03^	1.10 × 10^–03^	^b^
Transferase activity	1.20 × 10^–03^	1.50 × 10^–04^	6.50 × 10^–04^	1.70 × 10^–05^	1.80 × 10^–05^
Misfolded protein binding	1.80 × 10^–03^	5.30 × 10^–04^	2.50 × 10^–03^	^b^	^b^
Phosphoserine residue binding	5.30 × 10^–03^	^b^	^b^	7.40 × 10^–04^	^b^
GTP binding	9.20 × 10^–03^	5.40 × 10^–03^	^b^	^b^	^b^
Structural constituent of chromatin	^b^	^b^	2.50 × 10^–03^	^b^	2.80 × 10^–03^
Cadherin binding	^b^	^b^	6.40 × 10^–03^	^b^	^b^
Protein heterodimerization activity	^b^	^b^	^b^	^b^	2.50 × 10^–03^
Hydrolase activity	^b^	2.60 × 10^–03^	^b^	^b^	^b^
RNA binding	^b^	2.80 × 10^–03^	^b^	^b^	^b^
GTPase activity	^b^	3.80 × 10^–03^	^b^	^b^	^b^

a No molecular functions were found to be enriched in the bispecific class HCPs so a column representing it is not included in the table.

b MF not significantly enriched in the group.

c All_HCP: GO Term enrichment performed using all HCPs identified.

d Fc-fusion: GO Term enrichment of HCPs identified on the FC-fusion class products.

e IgG1: GO Term enrichment of HCPs identified on the IgG1 class products.

f IgG2: GO Term enrichment of HCPs identified on the IgG2 class products.

g IgG4: GO Term enrichment of HCPs identified on the IgG4 class products.

In total, REVIGO associated 13 GO Terms with 12 GO Term clusters. The interactive graph (Figure 5A) illustrates the degree of similarity between these GO Term clusters. Clusters are shown as bubbles where the color of the bubble represents the degree of enrichment (*p*-value, lighter = smaller value) and the size of the bubble represents the frequency of the term in the Gene Ontology Annotation (GOA) Database. Similar terms are linked by gray lines (edges) where the thickness of the edge correlates to the degree of similarity. Nine GO Term cluster groupings were identified, with the greatest similarity seen between “ATP-dependent protein folding chaperone” and “ATP-hydrolysis activity”. The tree map in Figure 5B shows high-level groupings of the GO Term clusters, represented by rectangles, with similar clusters grouped together and color coded. The size of each rectangle corresponds to the level of enrichment, with larger squares illustrating greater enrichment (i.e., smaller *p*-values). The 9 cluster groupings seen in Figure 5A were further grouped into 7 “super clusters” in Figure 5B.

**Figure 5:**
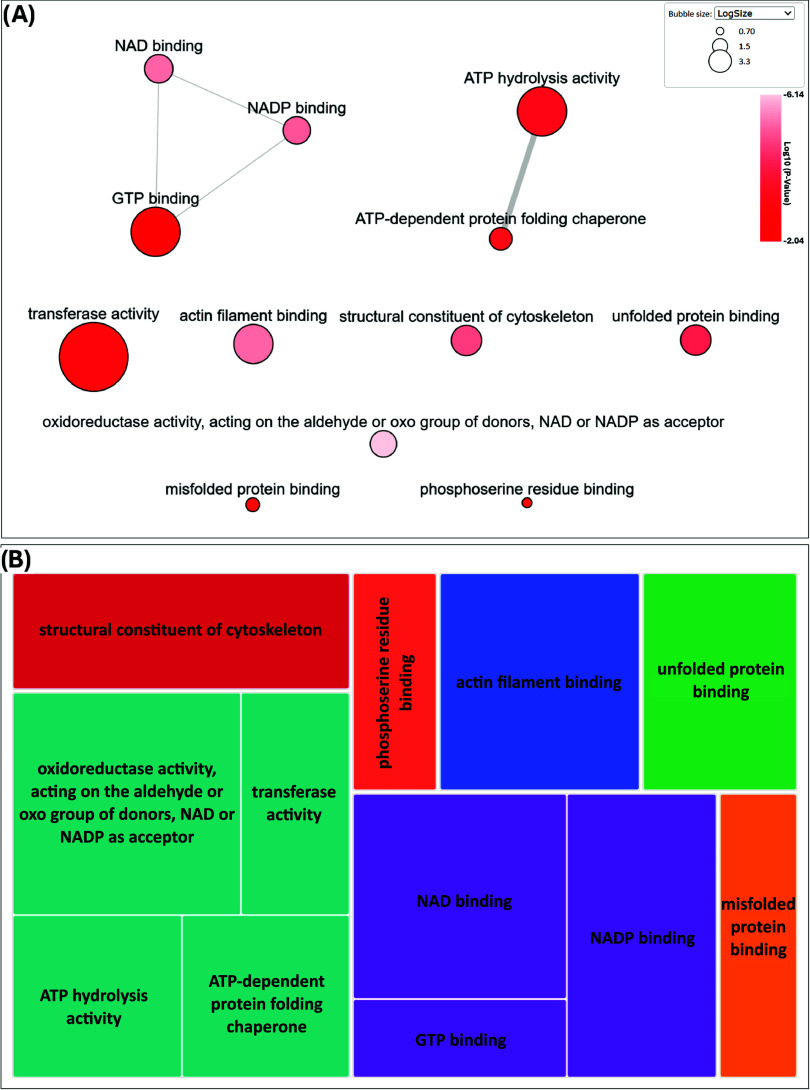
GO Term visualization of direct molecular functions associated with identified HCPs. Identifications were enriched using DAVID with the associated GO Terms visualized using REVIGO. (A) Graph demonstrating degree of similarity between GO Terms. Bubble color is associated with degree of enrichment (*p*-value) and size represents the frequency of term in the GOA database. Similar terms are linked by gray lines (edges) where edge thickness correlates to the degree of similarity. (B) Tree map showing high-level groupings of the input GO Terms, shown as rectangles. Rectangle size corresponds to level of enrichment, with larger squares illustrating greater enrichment (i.e., smaller *p*-values). Similar groups are clustered together and color coded.

We next performed GO Term enrichment on the HCPs associated with each product class to compare enriched MFs profiles across them. In DAVID 160, 91, 78, 51, and 38 gene IDs were associated with the Fc-fusion, IgG1, IgG2, IgG4, and bispecific groups in their respective analyses. “Catalytic activity” (47%, 76 gene IDs), “protein binding” (54%, 49 gene IDs), “small molecule binding” (51%, 40 gene IDs), “heterocyclic compound binding” (33%, 17 gene IDs), and “ribonucleoside triphosphate phosphatase activity” (24%, 9 gene IDs) had the most gene IDs associated with them for the Fc-fusion, IgG1, IgG2, IgG4, and bispecific groups when evaluating the MF_ALL analysis for each classification (Table S10). Analysis of the MF_DIRECT results revealed that while 15, 12, 9, and 8 MFs were significantly enriched in the Fc-fusion, IgG1, IgG2, and IgG4 classifications respectively, no MFs in the bispecific class were enriched below the B–H adjusted *p*-value ≤ 0.01 (Tables 1 and Table S11). However, five were found enriched below the B–H adjusted *p*-value ≤ 0.05 (Table S11).

A comparison of the Fc-fusion, IgG1, IgG2, and IgG4 groups revealed differences in MF enrichment between the product classifications with significantly enriched direct MFs (Table 1). Six MFs were found to be associated with all product classes. A majority of these were related to NAD functions, which play crucial roles in cellular metabolism. In some cases, such as with the MF “transferase activity”, the degree of enrichment was similar. However, in others such as “NADP binding” and “structural constituent of cytoskeleton”, the degree of enrichment between groups was quite varied. We additionally saw that each group had at least one instance of an enriched MF unique to its classification. For the IgG1, IgG2, and IgG4 classes, this was “cadherin binding”, “phosphoserine residue binding”, and “protein heterodimerization activity” respectively. However, for the Fc-fusion class five unique MFs were identified including “ATP hydrolysis activity”, “GTP binding”, GTPase activity”, “hydrolase activity”, and “RNA binding”. Furthermore, when performing GO Term enrichment analysis on the HCPs found to be unique to the Fc-fusion classification (Unique_Fc-fusion), “RNA binding” was found to be significantly enriched (Table S11). No MFs were found to be significantly enriched in the HCPs seen to be unique to any of the other product classes.

### Protein–Protein Interactions

Here we investigated protein–protein interactions between the HCPs identified, using STRING to perform functional protein association network analysis. The STRING database uses genomic, coexpression, and high-throughput experimental data along known protein complexes from curated sources to assess evidence of protein–protein interactions.^54^ From this assessment, interaction evidence scores are calculated on a scale of 0–1, with scores of 0.7 or greater signifying high confidence of a protein–protein interaction. Here all HCPs were mapped (Figure 6). In total 71 of the 236 HCPs (30.08%) were part of functional or physical association networks with interaction evidence scores greater than 0.7. Twelve HCP clusters were identified with the 2 most prominent clusters associated with the “proteosome” (7 HCPs) and “eukaryotic translation elongation” (5 HCPs) respectively (Figure 6). All other clusters were smaller, only associated with 2 or 3 HCPs. When exploring only physical protein complexes, the number of network connections reduced to only 4 clusters, demonstrating that according to STRING many of the protein–protein interactions seen are functional, but not physical in nature. We see that while the HCP associations in the “proteosome” cluster are mostly physical in nature, those involving Psmd9 and I79_012144 are not. All interactions in the “eukaryotic translation elongation” cluster were found not to be of a physical nature. The only other physical interactions are those of the HCPs comprising the “negative regulation of vesicle fusion” (2 HCPs), “UTP biosynthesis” (2 HCPs), and “AnxA2-p11 complex” (2 HCPs) clusters.

**Figure 6:**
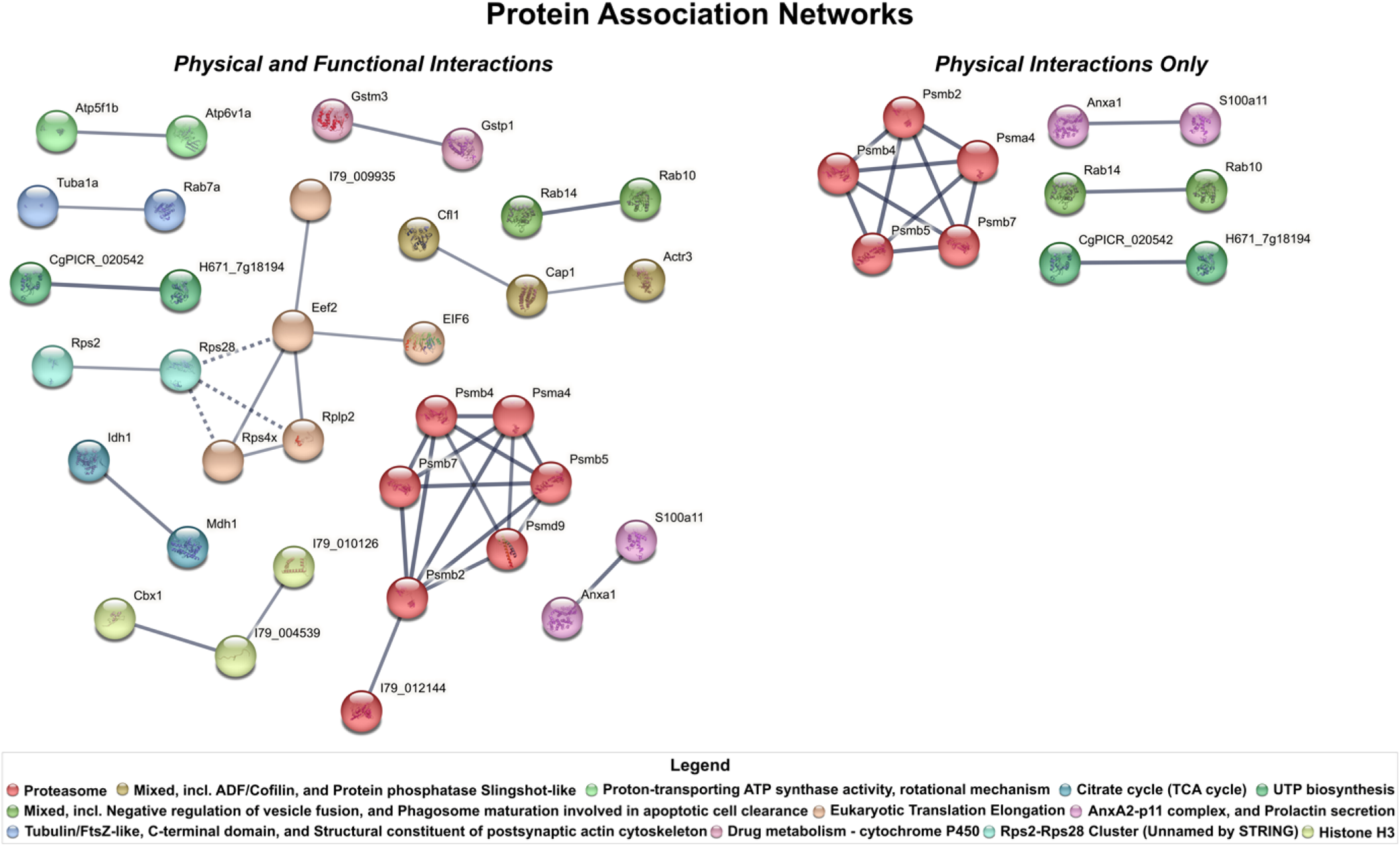
Protein association networks of identified HCPs across all products. Identified HCPs were subjected to protein–protein interaction analysis using the STRING database (version 12.0). Only associations with an interaction evidence score greater than 0.7 are displayed and the width of the edges (solid gray lines) reflects the interaction evidence. Node colors are assigned based on the protein clusters found in STRING database using MCL clustering. Interaction evidence between cluster edges is indicated using dotted gray lines. HCPs without any association are not displayed. Descriptions of the HCPs contained within these networks can be found in Table S12. (Left) Each node represents an HCP known to be part of a physical or functional network. (Right) Each node represents an HCP known to be part of a physical network only.

Evaluating protein–protein interactions for the HCPs identified in each product classification shows how certain interaction clusters are more associated with specific product classes. Protein interaction clusters were only found in the Fc-fusion, IgG1, and IgG2 product classes using the previously described parameters (Figure S6). The “AnxA2-p11 complex, and prolactin secretion” cluster was seen across all three product classes while the “histone H3” cluster was present in only the Fc-fusion and IgG1 classes. The “proteasome”, “eukaryotic translation elongation”, proton-transporting ATP synthase activity, rotational mechanism”, and “tubulin/FtsZ-like, C-terminal domain, and structural constituent of postsynaptic actin cytoskeleton” clusters were associated solely with the Fc-fusion class, with the “proteasome” cluster being the only cluster present in the network analysis of HCPs unique to the Fc-fusion products. The “mixed, incl. ADF/cofilin, and protein phosphatase slingshot-like” cluster was solely associated with the IgG2 class while an Eef2-Rps28 cluster was identified in the IgG1 class. However, this cluster was not seen when performing protein–protein interaction analysis on all the identified HCPs (Figure 6). There, Eef2 was associated with “eukaryotic translation elongation” while Rps28 had formed a cluster with Rps2, an HCP not seen in the IgG1 product class.

### Detection of Potential “High-Risk” HCPs

Understanding which HCP impurities are present on a product is crucial as certain HCPs are considered more problematic than others due to their potential to impact product efficacy and patient safety.^4^ Due to the importance of their potential presence, we searched for potentially high-risk HCPs in all the products analyzed. Our main reference was the high-risk HCPs identified by the BPDG in Jones et al.^56^ This is a resource intended to inform that the presence of such HCPs has the potential to cause a human safety risk. However, it does not indicate that their presence will result in adverse patient outcomes. It must be noted this list is regularly updated by the Biophorum, but the most up-to-date version is only available on a subscription basis (https://www.biophorum.com/host-cell-protein-data-platform/) and thus our findings are based on a potentially outdated data set. Still, we were able to identify HCPs explicitly stated as high-risk by Jones et al. (Figure 7A). Additionally, we detected HCPs that were determined by DAVID to be functionally similar or identical to said high-risk HCPs but listed in Uniprot as unique proteins (Figure 7A). Given our search parameters required two unique peptides for a valid identification, we included these HCPs in our results but recognize that without further study these HCPs cannot be explicitly considered high-risk. To differentiate between these HCPs, in this manuscript, only HCPs explicitly identified in Jones et al. (I.e. same Uniprot accession number) are referred to as potentially high-risk HCPs while those functionally similar to the high-risk HCPs are referred to as potentially problematic HCPs.^56^ Ideally, we would have used a resource such as CHOPPI, a tool to analyze the immunogenicity risk of CHO derived HCPs, to further assess the potential immunogenicity risk of these potentially problematic HCPs.^77^ However, as far as we can tell CHOPPI is no longer publicly accessible, with its function now appearing to be incorporated into EpiVAX’s ISPRI-HCP workflow, accessible on a fee-for-service basis (https://epivax.com/insilico-tx/ispri-hcp/). Finally, we included histones in our list of potentially problematic HCPs since they can play a role in transporting more harmful HCPs through downstream purification processes.^57^

**Figure 7:**
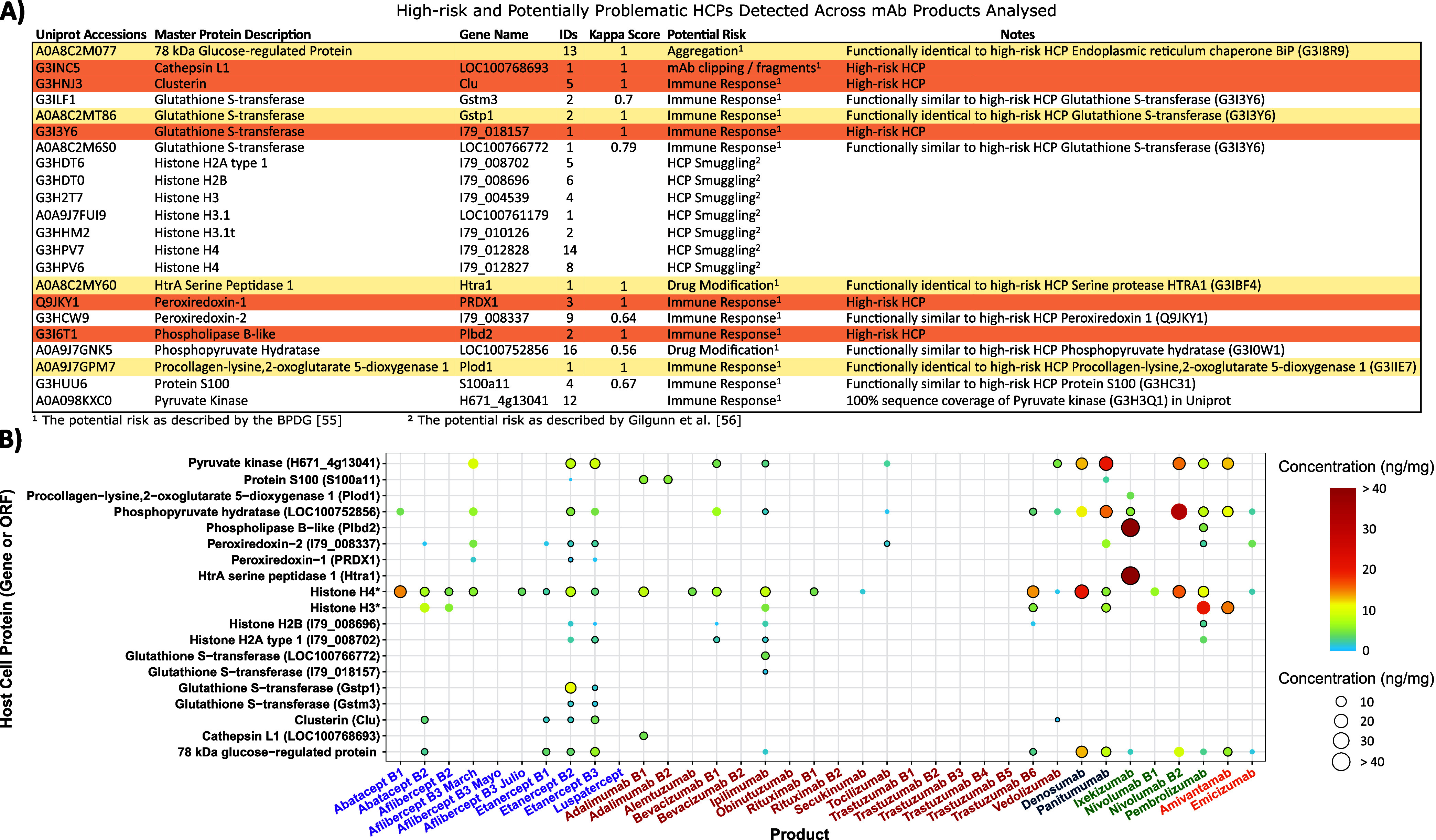
Potential high-risk and problematic HCPs detected across all protein biotherapeutics analyzed. (A) Table describing the potential high-risk and problematic HCPs identified and their potential risk. Rows in orange are HCPs determined as high-risk by the BPDG.^56^ Rows in gold are HCPs considered by DAVID to be functionally identical to high-risk HCPs identified by the BPDG, just under a different accession number. (B) Dot plot of potentially high-risk and problematic HCP occurrences across all products analyzed. Dot size and color correspond to HCP concentration, though HCP concentrations ≥40 ng/mg are all shown as the same size and color. HCP abundances were determined using Hi3 quantitation, which requires 3 unique peptides. Where HCPs were identified with only 2 unique peptides, abundances were estimated using those 2 peptides only. Product names in table are ordered and colored by class: Fc-fusion–Purple, IgG1–Red, IgG2–Blue, IgG4–Green, Bispecific–Orange.

In total 22 potentially high-risk or potentially problematic HCPs were identified across all the protein biotherapeutics studied. Five of these HCPs were found to be high risk by the BPDG when searching for their Uniprot accession number (rows highlighted in orange in Figure 7A). Accession numbers corresponding to an additional four HCPs were considered functionally identical to high-risk HCPs identified by the BPDG, just under a different accession number (rows highlighted in gold in Figure 7A). Three HCPs were found to be functionally related to glutathione s-transferase (G3I3Y6; GST) and one apiece functionally related to protein S100 (G3HC31), peroxiredoxin-1 (Q9JKY1; PRDX1), and phosphopyruvate hydratase (G3I0W1) respectively. The accession number A0A098 KXC0, corresponding to a pyruvate kinase HCP, was also identified, but it was not recognized by DAVID. Aligning the sequence of this HCP with that of the pyruvate kinase considered a high-risk HCP by the BPDG revealed that the identified HCP is considerably larger than the one considered as high-risk by the BPDG. However, 100% of the sequence of the high-risk HCP did align with our identified pyruvate kinase HCP, so we considered it to be potentially problematic. Multiple glutathione s-transferase and histone H4 HCPs were identified. In each case the respective proteins were associated with different genes, so each identification was considered unique. These identified high-risk HCPs have a variety of potential risks including product aggregation, mAb clipping, drug modification, potential to cause an immune response according to the BPDG.^56^ Additionally, seven histone HCPs were detected.

There is a wide distribution of these potentially high-risk or problematic HCPs across the protein biotherapeutics analyzed, both in terms of concentration and number of products detected in, as illustrated by the dot plot in Figure 7B. In total potentially high-risk or problematic HCPs were detected 113 times across all the protein biotherapeutics analyzed. Unsurprisingly, the total number of potentially high-risk or problematic HCPs detected on any specific product often correlated with the total number of HCPs identified, with etanercept batches 2, and 3 containing the most potentially high-risk or problematic HCPs. Histones were most ubiquitous, detected in over 63% (23) of all products at varying concentrations. Phosphopyruvate hydratase, 78 kDa glucose-regulated protein, pyruvate kinase, and peroxiredoxin-2 were also readily present, identified in over 44% (16), 36% (13), 33% (12), and 25% (9) of the products analyzed, respectively. Conversely, five potentially high-risk or problematic HCPs were found to be present in only one protein biotherapeutic. These consisted of cathepsin L1 (G3INC5), two glutathione s-transferases (G3I3Y6 and A0A8C2M6S0), HtrA serine peptidase 1 (A0A8C2MY60; HTRA1), and procollagen-lysine,2-oxoglutarate 5-dioxygenase 1 (A0A9J7GPM7). Of the 113 times a potentially high-risk HCP was detected, 41 (36%) could not be quantified using Hi3 quantitation as they were only detected with two unique peptides (Dots with no edges in Figure 7B). Here their concentrations were approximated using only the two unique peptides used for identification given the significance of their presence in the samples. Generally, the individual concentrations of high-risk or problematic HCPs followed the concentration trends of the quantifiable HCPs discussed in Benchmarking Orbitrap Astral MS Performance Using NISTmAb section. 95% (107 HCPs), 83% (94 HCPs), and 10% (11 HCPs) had individual concentrations below 20 ng/mg, 10 ng/mg and 1 ng/mg, respectively. Of the six instances where an individual concentration was greater than 20 ng/mg, four of them had individual concentrations no greater than 33 ng/mg. However, two potentially high-risk or problematic HCPs, HTRA1 and Phospholipase B-like (PLBL2), were found at very high individual concentrations of 134.6 ng/mg and 95.5 ng/mg, respectively.

Grouping the products by classification enabled comparison of potentially high-risk or problematic HCP profiles across product classes. The average concentration of a detected potentially high-risk or problematic HCP on an Fc-fusion product was 47 ng/mg, with individual high-risk HCPs detected at concentrations below 10 ng/mg and 5 ng/mg respectively, 95% and 69% of the times. For the IgG1 products the average concentration of a detected high-risk HCP was 3.7 ng/mg with individual high-risk HCPs detected at concentrations below 10 ng/mg and 5 ng/mg 97% and 76% of the times, respectively. This was true even if the total number of potentially high-risk or problematic HCPs on a product was relatively high, as was the case with etanercept batches 2 and 3, as well as ipilimumab. However, with the IgG2 products, the average concentration of a detected potentially high-risk or problematic HCP was 11.2 ng/mg and only 45% of the detected potential high-risk or problematic HCPs have concentrations below 10 ng/mg. While the concentrations of these potentially high-risk or problematic HCPs above 10 ng/mg was still low, between 11 and 21 ng/mg, they were generally more concentrated in the IgG2 products compared to the Fc-fusion and IgG1 products. Of the seven times a high-risk HCP was found to have a concentration greater than 20 ng/mg, four of those instances occurred on IgG4 products, including the previously mentioned HTRA1 and PLBL2 detected at concentrations of 134.6 ng/mg and 95.5 ng/mg, respectively. Interestingly, both instances were found on the same product, ixekizumab. HTRA1 can cause modifications to the drug product^56^ while PLBL2 is associated with immune response and is understood to bind to IgG4 molecules.^64^ PLBL2 is also thought to degrade polysorbates used in product formulation,^78^ which can decrease product stability, though a recent study has suggested that this may not be the case.^79^ As stated previously, this was the only time HTRA1 was detected. Moreover, PLBL2 was detected in one other instance, also an IgG4 product, but at a significantly lower concentration, 5.0 ng/mg. This all resulted in the average concentration of detected potentially high-risk or problematic HCPs on IgG4 products being 20.27 ng/mg, despite over 63% having concentrations below 10 ng/mg. Finally, the bispecific products generally had low concentrations, as the average concentration of a detected potentially high-risk or problematic HCP was 6.81 ng/mg and nearly 63% had concentrations below 10 ng/mg. While the cohort analyzed is not exhaustive, these findings, along with others^80^ suggest that tailoring purification strategies based on IgG product class could be beneficial for reducing individual product HCP levels.

Of the HCPs identified as potentially high-risk or potentially problematic, 11 are potential immunogenic risks as described by Jones et al. or based on their functional similarities to HCPs identified as immunogenic risks by Jones et al.^56^ These include the high-risk HCPs clusterin, GST, PRDX1, and phospholipase B-like (PLBL2) as well as 3 HCPs functionally related to GST, 1 HCP functionally related to PRDX1, an HCP functionally identical to procollagen-lysine, 2-oxoglutarate 5-dioxygenase 1, an HCP functionally related to protein S100, and a pyruvate kinase. While it is beyond the purview of this study to assess the immunogenicity of each of these HCPs, it is worth reiterating that in silico tools such as the now defunct CHOPPI, EpiVAX’s fee-for-service ISPRI-HCP workflow, and the IEDB (https://www.iedb.org/) can assist in determining potential HCP immunogenicity risks. Furthermore, it is the de Zafra risk assessment framework which provides the most comprehensive methodology for determining adverse outcomes associated with the presence of HCPs.^4,12^ This framework focuses on how biological activity and immunogenic risk factors are influenced by the identity of the HCP, ones experience with said HCP, the therapeutic dosage and dosage frequency, the therapeutic mechanism of action, clinical indication/population, and the treatment duration. In using such criteria, this framework allows for the assessment of potential adverse outcomes due to HCP impurities on a case-by-case basis where factors such as HCP concentration are only a part of a more holistic approach toward patient safety.

## Conclusions

The development of the Orbitrap Astral MS has helped advance proteomic analysis of complex sample matrices with significant improvements in analysis times, protein identifications, or both. In this study the Orbitrap Astral MS was utilized to perform HCP analysis on a wide range of protein biotherapeutics including IgG1, IgG2, IgG4, bispecific and Fc-fusion products. The speed and sensitivity of the Astral MS analyzer facilitated the use of short LC gradients for fast analysis of large sample cohorts without sacrificing HCP detection. An analysis time that would have required weeks using other instrumentation was reduced to days using the Orbitrap Astral MS. Leveraging DIA analysis and advanced data processing algorithms, it was possible to detect and quantify HCPs at sub 1 ng/mg concentrations, with a great majority of HCPs detected at sub 10 ng/mg levels. This all without requiring time-consuming sample preparation strategies to minimize the presence of protein biotherapeutic peptides and reduce dynamic range before analysis. Even when hundreds of HCPs were present in a single product, Orbitrap Astral MS was still able to detect and quantify HCPs at sub 1 ng/mg levels. The ability to perform such low-level quantitation is especially important when trying to detect potentially high-risk HCPs, as such HCPs can have negative impacts on product quality and patient outcomes even at low levels. The study demonstrates that the Orbitrap Astral MS can be used to monitor trace-level HCPs, even in a high throughput environment.

## Supplementary Material











## Data Availability

The mass spectrometry proteomics data have been deposited to the ProteomeXchange Consortium via the PRIDE partner repository with the data set identifier PXD067004 and 10.6019/PXD067004.

## Notes

Benchmarking quantified HCPs identified on NISTmAb in our study versus HCPS detected in literature (Figure S1); comparing NISTmab HCPs identified using native digestion and SP3 digestion protocol (Figure S2); heatmap profile of HCPs identified on all protein biotherapeutic products (Figure S3); physico-chemical properties of identified HCPs (Figure S4); HCP physicochemical properties in relation to HCP concentration (Figure S5), and association networks of HCPs identified in each product classification (Figure S6) (PDF)
Full descriptions of the protein biotherapeutics analyzed, the LC and MS parameters used for analysis, and the publicly available “high-risk” host cell proteins as determined by the BPDG (Tables S1–S3 and S5) (XLSX)
HCP identifications for each protein biotherapeutic product analyzed after application of primary validation filters (Table S4­(A–JJ)) (XLSX)
Additional information on the IgG-like, desmosome, and keratinization associated proteins excluded during data filtering as well as a full list of all the host cell proteins identified in our analysis and all instances where they were quantified (Tables S6–S9) (XLSX)
Descriptions of the molecular functions enriched in DAVID for each analysis performed as well as the STRING identifiers/descriptions related to the HCP association networks illustrated in Figure 6 (Tables S10–S12) (XLSX)

## ∥

School of Medicine, University of Limerick, Limerick V94 T9PX, Ireland
