# Supplementary material for: Quantitative Host Cell Protein Analysis of Antibody-Based Protein Therapeutics Using the Orbitrap Astral Mass Spectrometer
Source: J Am Soc Mass Spectrom. 2026 Apr 17;37(5):1077–94. doi: 10.1021/jasms.5c00272 (PMC13154345; doi:10.1021/jasms.5c00272)
Supplement: Supplementary file 1 [file js5c00272_si_001.pdf]

Supporting Information

Quantitative Host Cell Protein Analysis of Antibody-Based Protein Therapeutics using the Orbitrap Astral Mass Spectrometer

Josh Smith[1], Aaron Richardson[1], Corentin Beaumal[1], Marina Ainciburu[1,2], Sara Carillo[1], Anna Pashkova[3] ,Tabiwang N. Arrey[3], Nicolaie E. Damoc[3], Colin Clarke[1,4] and Jonathan Bones[1,4*]

[1]National Institute for Bioprocessing Research and Training, Foster Avenue, Mount Merrion, Blackrock, Co. Dublin, Ireland, A94 X099.

[2]Current address: School of Medicine, University of Limerick, Limerick, Ireland, V94 T9PX.

[3]Thermo Fisher Scientific GmbH, Hannah-Kunath Strasse 11, 28199 Bremen, Germany.

[4]School of Chemical and Bioprocess Engineering, University College Dublin, Belfield, Dublin 4, Ireland, D04 V1W8.

**Corresponding author*: Jonathan Bones, email: jonathan.bones@nibrt.ie, tel: +353 1215 8100, fax: +353 1215 8116.

List of Supporting Information

Table of Contents

Supplementary Figures
Figure S1: Benchmarking quantified HCPs identified on NISTmAb in our study versus HCPS detected in literature
Figure S2: Comparing NISTmab HCPs identified using native digestion and SP3 digestion
Figure S3: Heatmap profile of HCPs identified on all protein biotherapeutic products
Figure S4: Physico-chemical properties of identified HCPs
Figure S5: HCP physico-chemical properties in relation to HCP concentration
Figure S6: Association networks of HCPs identified in each product classification
Supplementary Tables
Table S1: Protein biotherapeutic products tested during HCP analysis
Table S2: Vanquish™ Neo Nano-LC separations method for mAb based product HCP analysis
Table S3: Orbitrap™ Astral™ MS settings for mAb based product HCP analysis
Table S4: HCP identifications for each protein biotherapeutic product analysed after application of primary validation filters
(A) HCPs identified on abatacept batch 1 after application of primary validation filters
(B) HCPs identified on abatacept batch 2 after application of primary validation filters
(C) HCPs identified on adalimumab batch 1 after application of primary validation filters
(D) HCPs identified on adalimumab batch 2 after application of primary validation filters
(E) HCPs identified on aflibercept batch 2 after application of primary validation filters
(F) HCPs identified on aflibercept batch 3 Julio after application of primary validation filters
(G) HCPs identified on aflibercept batch 3 March after application of primary validation filters
(H) HCPs identified on aflibercept batch 3 Mayo after application of primary validation filters
(I) HCPs identified on alemtuzumab after application of primary validation filters
(J) HCPs identified on amivantamab after application of primary validation filters
(K) HCPs identified on bevacizumab batch 1 after application of primary validation filters
(L) HCPs identified on bevacizumab batch 2 after application of primary validation filters
(M) HCPs identified on denosumab after application of primary validation filters
(N) HCPs identified on emicizumab after application of primary validation filters
(O) HCPs identified on etanercept batch 1 after application of primary validation filters
(P) HCPs identified on etanercept batch 2 after application of primary validation filters

(Q) HCPs identified on etanercept batch 3 after application of primary validation filters
(R) HCPs identified on ipilimumab after application of primary validation filters
(S) HCPs identified on ixekizumab after application of primary validation filters
(T) HCPs identified on luspatercept after application of primary validation filters
(U) HCPs identified on nivolumab batch 1 after application of primary validation filters
(V) HCPs identified on nivolumab batch 2 after application of primary validation filters
(W) HCPs identified on obinutuzumab after application of primary validation filters
(X) HCPs identified on panitumumab after application of primary validation filters
(Y) HCPs identified on pembrolizumab after application of primary validation filters
(Z) HCPs identified on Rituximab batch 1 after application of primary validation filters
(AA) HCPs identified on Rituximab batch 2 after application of primary validation filters
(BB) HCPs identified on secukinumab after application of primary validation filters
(CC) HCPs identified on tocilizumab after application of primary validation filters
(DD) HCPs identified on trastuzumab batch 1 after application of primary validation filters
(EE) HCPs identified on trastuzumab batch 2 after application of primary validation filters
(FF) HCPs identified on trastuzumab batch 3 after application of primary validation filters
(GG) HCPs identified on trastuzumab batch 4 after application of primary validation filters
(HH) HCPs identified on trastuzumab batch 5 after application of primary validation filters
(II) HCPs identified on trastuzumab batch 6 after application of primary validation filters
(JJ) HCPs identified on vedolizumab after application of primary validation filters
**Table S5: Publicly available list of potentially high-risk HCPs as determined by the BPDG**
**Table S6: Ig-like proteins identified across the analysed protein biotherapeutic products**
**Table S7: Desmosome and keratinization associated proteins**
**Table S8: All HCPs identified during analysis**
**Table S9: All instances where an HCP was quantified during analysis**
**Table S10: All molecular functions enriched in DAVID for each analysis performed**
**Table S11: Direct molecular functions enriched in DAVID for each analysis performed**
**Table S12: STRING identifiers and descriptions of the HCPs within the mapped protein association networks illustrated in Figure 7**
References

Supplementary Figures

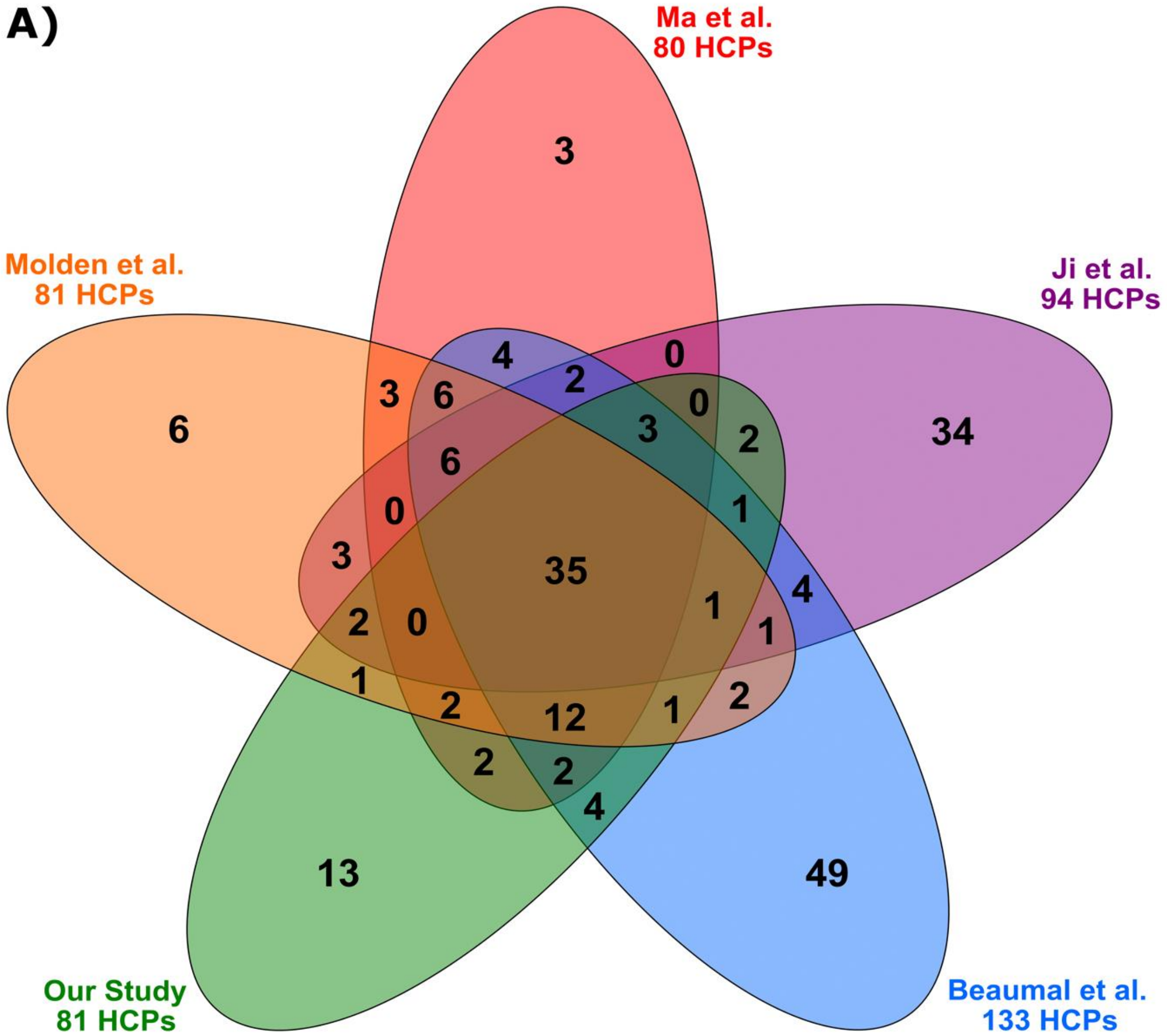

Figure S1: Benchmarking quantified HCPs identified on NISTmAb in our study versus HCPS detected in literature. Venn diagram comparing quantified HCPs identified on NISTmAb in our study (Green) versus HCPS detected in Molden et al.[1] (Orange), Ma et al. (44 min)[2] (Red), Ji et al.[3] (Purple) and Beaumal et al.[4] (Blue). For all studies native digestion of NIST was performed based on the procedure described in Huang et al.[5]. Data from all studies was acquired using nanoLC-MS/MS with the exception of Molden et al. and Ji et al. whose studies utilized ultra performance LC-MS/MS.

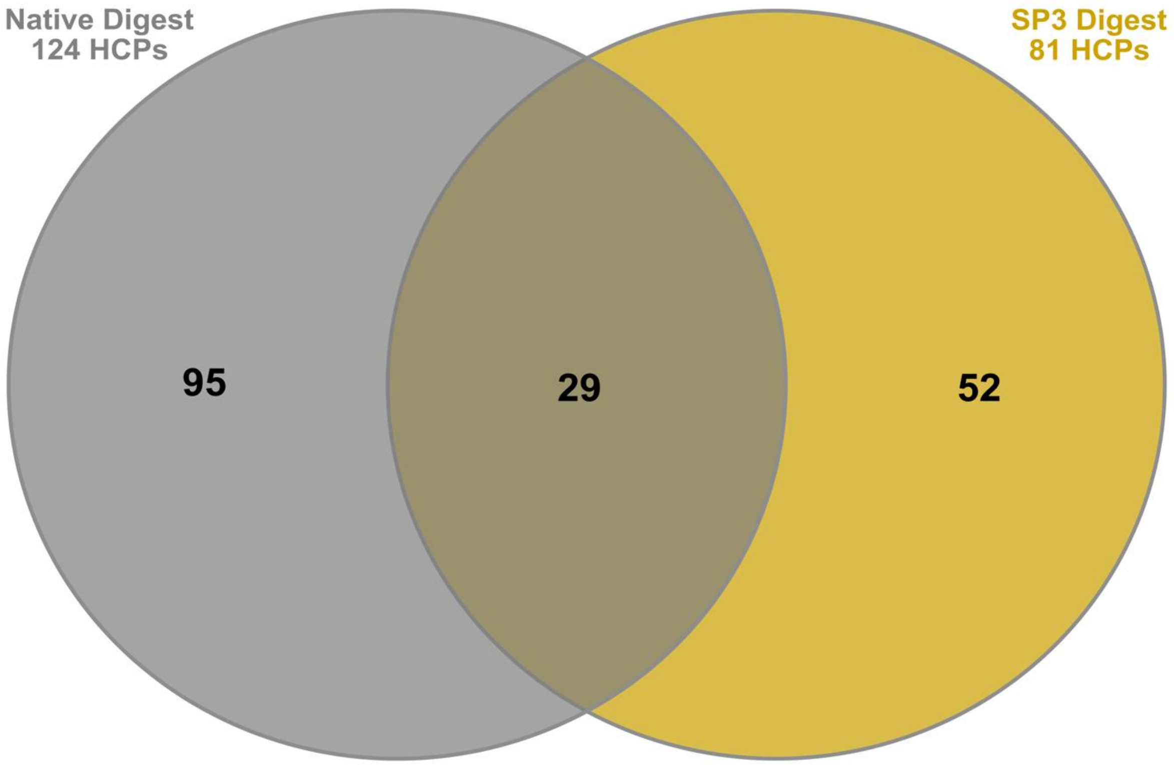

Figure S2: Comparing NISTmab HCPs identified using native digestion and SP3 digestion. Venn diagram showing overlap of NISTmAb HCPs identified in the native digest versus those detected using the SP3 digest.

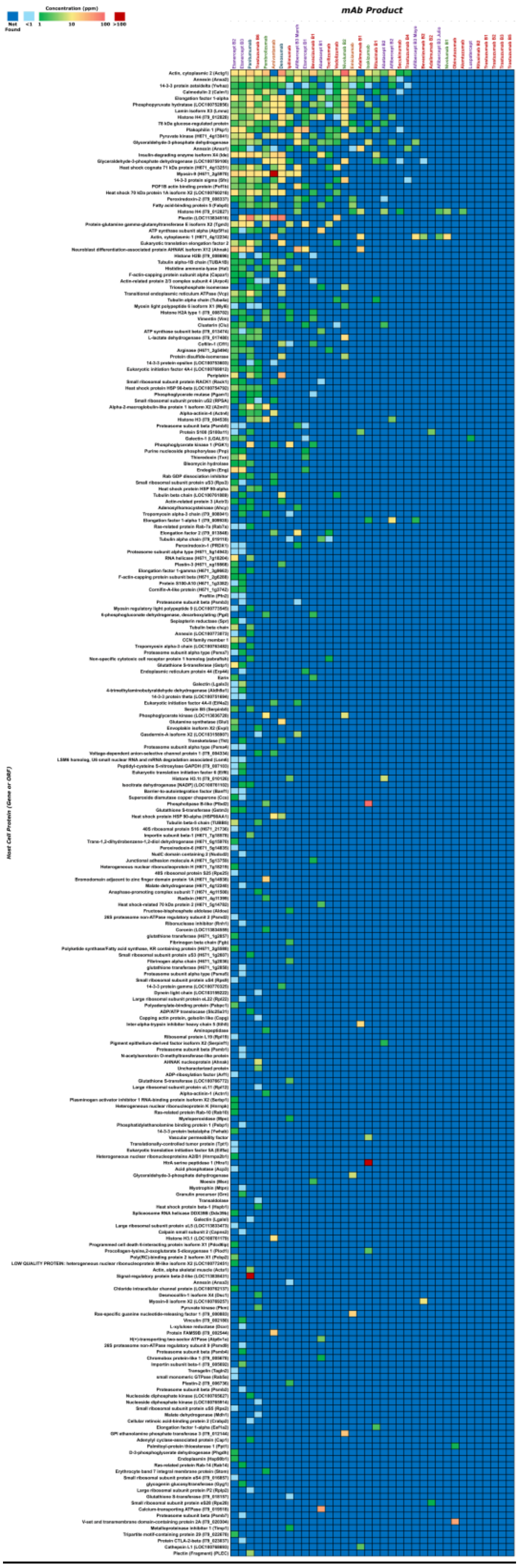

Figure S3: Heatmap profile of HCPs identified on all protein biotherapeutic products. Columns represent mAb products ranked by decreasing number of HCPs detected. Product names are coloured by class: Fc-fusion – Purple, IgG1 – Red, IgG2 – Blue, IgG4 – Green, Bispecific – Orange. Rows detail each HCP identified ordered by occurrence across mAb products. HCP abundances were determined using Hi3 quantitation, requiring 3 unique peptides. Where HCPs were identified with only 2 unique peptides, abundances were estimated using those 2 peptides only. Colours signify HCP abundances ranging from 0 ppm (dark blue, not detected) to >100 ppm (dark red). HCPs detected with abundances below 1ppm are highlighted in light blue.

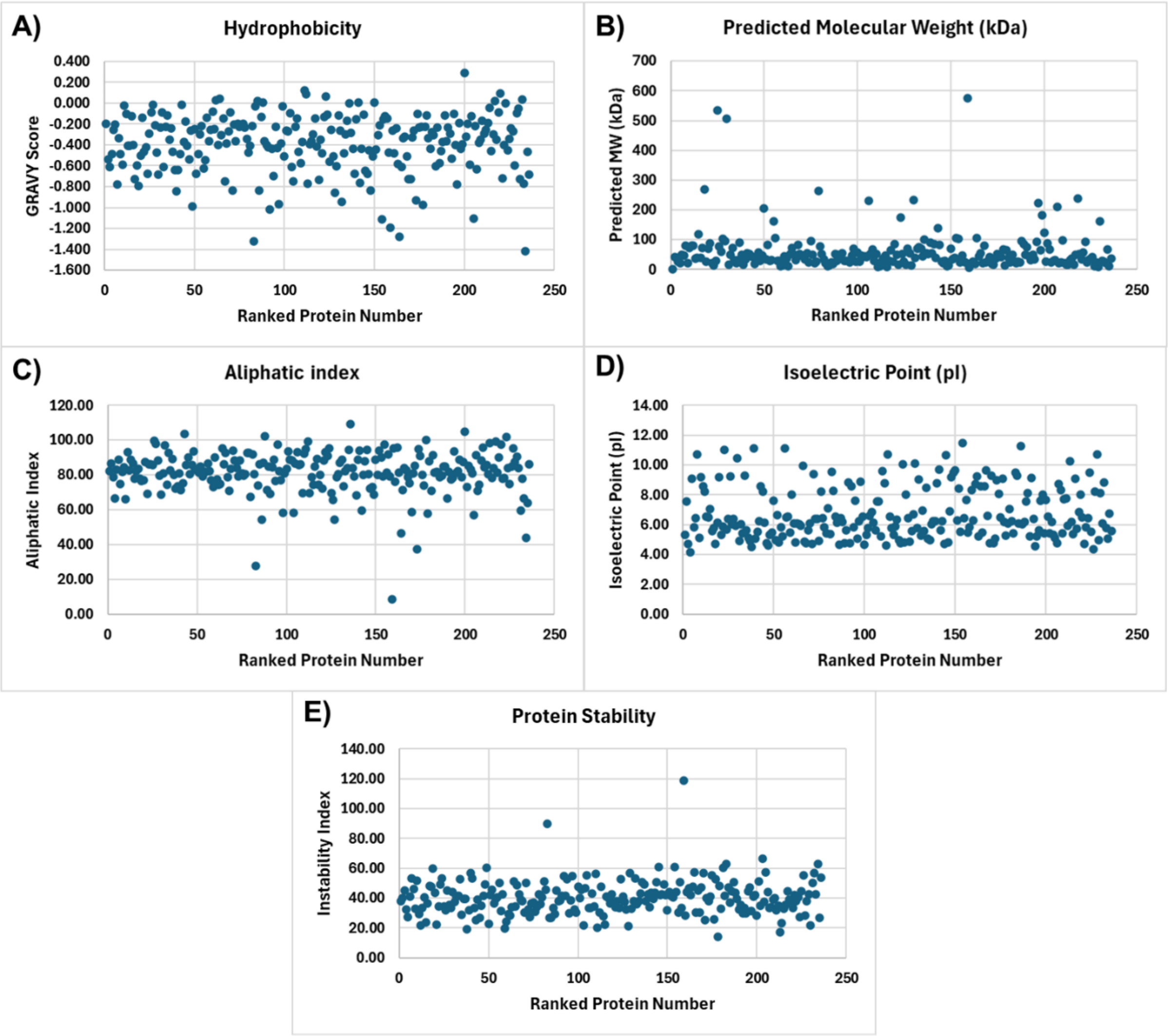

F)

	Hydrophilic	MW < 100 kDa	MW < 50 kDa	AI > 60	AI > 80	pI	Stable
# of HCPs	223	211	142	222	151	157	120
% of HCPs	94	89	60	94	64	66	51

Figure S4: Physico-chemical properties of identified HCPs. The relationship of the physico-chemical properties associated with the identified HCPs was explored using ProtParam (https://web.expasy.org/protparam/). **A)** Hydrophobicity. An HCP with a Grand Average of Hydropathy (GRAVY) score < 0 is considered hydrophilic. **B)** Predicted Molecular Weight. **C)** Aliphatic Index. The aliphatic index is an indicator of a protein's thermal stability with higher values suggesting greater thermostability at high temperatures. **D)** Isoelectric point. **E)** Protein Stability. Protein stability was determined using the instability index in ProtParam. An HCP with an instability index value < 40 is predicted to be stable, while an HCP with a value > 40 is predicted to be unstable. **F)** Table describing the number of HCPs associated with the evaluated protein physico-chemical properties. MW indicates molecular weight and AI indicates aliphatic index.

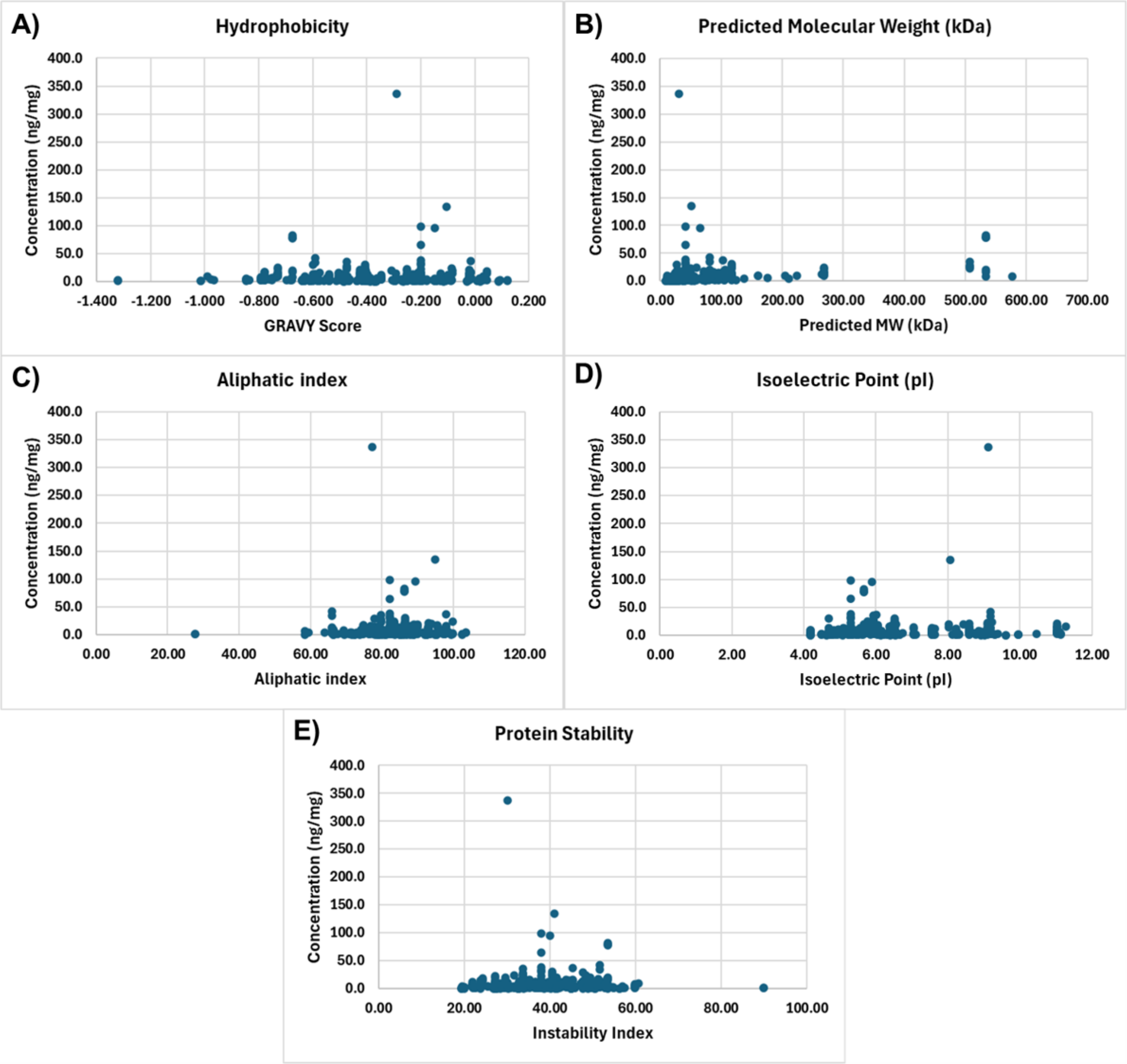

Figure S5: HCP physico-chemical properties in relation to HCP concentration. The relationship between physico-chemical properties and HCP concentration is explored by graphing the concentration any quantified HCP against their physico-chemical properties found using ProtParam (https://web.expasy.org/protparam/). All instances where an HCP was quantified were considered, so the same HCP can appear multiple times in these graphs. **A)** Hydrophobicity. An HCP with a Grand Average of Hydropathy (GRAVY) score < 0 is considered hydrophilic. **B)** Predicted Molecular Weight. **C)** Aliphatic Index. The aliphatic index is an indicator of a protein's thermal stability with higher values suggesting greater thermostability at high temperatures. **D)** Isoelectric point. **E)** Protein Stability. Protein stability was determined using the instability index in ProtParam. An HCP with an instability index value < 40 is predicted to be stable, while an HCP with a value > 40 is predicted to be unstable.

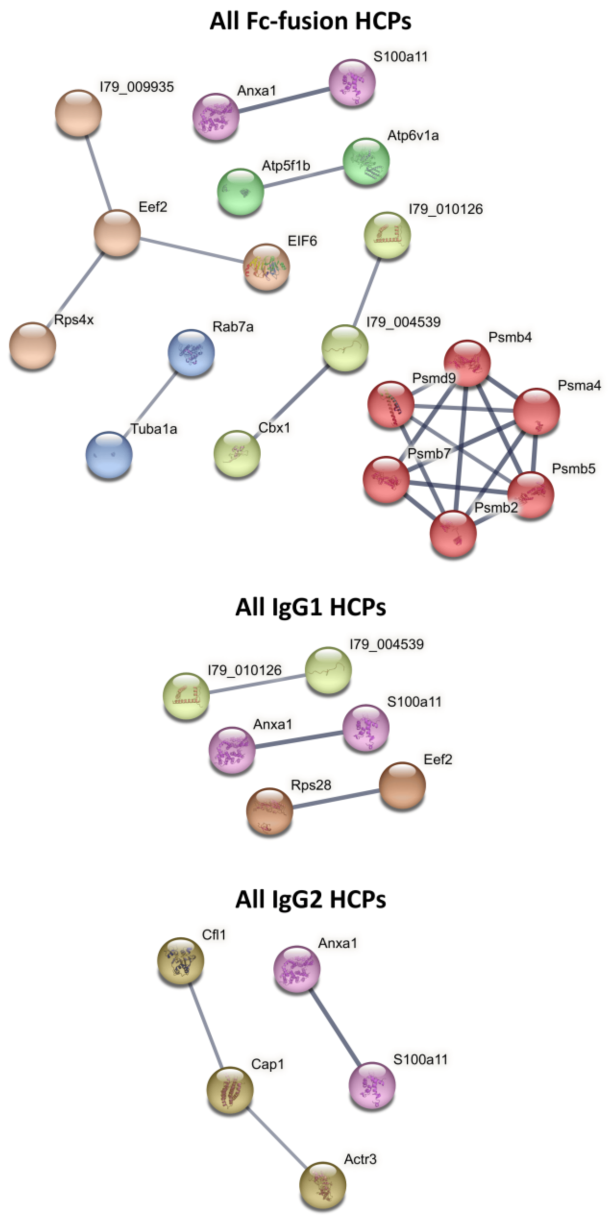

Legend

- **Proteasome**
- **Eukaryotic Translation Elongation**
- **Eef2-Rps28 Cluster (Unnamed by STRING)**
- **Mixed, incl. ADF/Cofilin, and Protein phosphatase Slingshot-like**
- **Histone H3**
- **Proton-transporting ATP synthase activity, rotational mechanism**
- **Tubulin/FtsZ-like, C-terminal domain, and Structural constituent of postsynaptic actin cytoskeleton**
- **AnxA2-p11 complex, and Prolactin secretion**

Gene name	Protein identifier	Protein description
Psma4	10029.G3H0C2	Proteasome subunit alpha type
Psmb2	10029.G3H9V0	Proteasome subunit beta
Psmb4	10029.G3H303	Proteasome subunit beta
Psmb5	10029.G3HRD9	Proteasome subunit beta
Psmb7	10029.G3I9D3	Proteasome subunit beta
Psmd9	10029.G3H256	26S proteasome non-ATPase regulatory subunit 9
EIF6	10029.G3HAQ2	Eukaryotic translation initiation factor 6
Rps4x	10029.G3I0H2	40S ribosomal protein S4
I79_009935	10029.G3HH39	Elongation factor 1-alpha 1
Eef2	10029.G3HSL4	Elongation factor 2
Rps28	10029.G3I5R2	40S ribosomal protein S28-like protein
Actr3	10029.G3GZE6	Actin-related protein 3
Cap1	10029.G3HN88	Adenylyl cyclase-associated protein
Cfl1	10029.G3IDM2	Cofilin-1
Cbx1	10029.G3H5T7	Chromobox protein-like 1
I79_004539	10029.G3H2T7	Histone H3
I79_010126	10029.G3HHM2	Histone H3.1t
Atp5f1b	10029.G3HRN0	ATP synthase subunit beta
Atp6v1a	10029.G3H066	V-type proton ATPase catalytic subunit A
Rab7a	10029.G3HID1	Ras-related protein Rab-7a
Tuba1a	10029.G3I6I6	Tubulin alpha chain
Anxa1	10029.G3I5L3	Annexin
S100a11	10029.G3HUU6	Protein S100

Figure S6: Association networks of HCPs identified in each product classification. Protein-protein interaction analysis was performed using the STRING database (version 12.0). Each node represents an HCP known to be part of a physical or functional network. Only associations with an interaction evidence score greater than 0.7 are displayed and the width of the edges (solid grey lines) reflects the interaction evidence. Node colours are assigned based on the protein clusters found in STRING database using MCL clustering. HCPs without any association are not displayed. Networks for HCPs identified on all Fc-Fusion products (All Fc-fusion HCPs), all IgG1 products (All IgG1 HCPs), and all IgG2 products (All IgG2 HCPs) were mapped. No clusters for the HCPs found on the IgG4 or Bispecific products were identified following the mapping criteria used.

Supplementary Tables

All supplementary tables can be found in the additional Excel spreadsheets

<u>**References**</u>

(1) Molden, R.; Hu, M.; Yen, E. S.; Saggese, D.; Reilly, J.; Mattila, J.; Qiu, H.; Chen, G.; Bak, H.; Li, N. Host cell protein profiling of commercial therapeutic protein drugs as a benchmark for monoclonal antibody-based therapeutic protein development. *MAbs* **2021**, *13* (1), 1955811. DOI: 10.1080/19420862.2021.1955811.
(2) Ma, J.; Kilby, G. W. Sensitive, Rapid, Robust, and Reproducible Workflow for Host Cell Protein Profiling in Biopharmaceutical Process Development. *Journal of proteome research* **2020**, *19* (8), 3396-3404. DOI: 10.1021/acs.jproteome.0c00252.
(3) Ji, Q.; Sokolowska, I.; Cao, R.; Jiang, Y.; Mo, J.; Hu, P. A highly sensitive and robust LC-MS platform for host cell protein characterization in biotherapeutics. *Biologicals* **2023**, *82*, 101675. DOI: 10.1016/j.biologicals.2023.101675.
(4) Beaumal, C.; Beck, A.; Hernandez-Alba, O.; Carapito, C. Advanced mass spectrometry workflows for accurate quantification of trace-level host cell proteins in drug products: Benefits of FAIMS separation and gas-phase fractionation DIA. *Proteomics* **2023**, *23* (16), e2300172. DOI: 10.1002/pmic.202300172.
(5) Huang, L.; Wang, N.; Mitchell, C. E.; Brownlee, T.; Maple, S. R.; De Felippis, M. R. A Novel Sample Preparation for Shotgun Proteomics Characterization of HCPs in Antibodies. *Analytical chemistry* **2017**, *89* (10), 5436-5444. DOI: 10.1021/acs.analchem.7b00304.
